# Analysis of Uncertainty and Sensitivity in Tailings Dam Breach-Runout Numerical Modelling

**DOI:** 10.1007/s10230-024-00970-w

**Published:** 2024-02-21

**Authors:** Negar Ghahramani, Daniel A. M. Adria, Nahyan M. Rana, Marcelo Llano-Serna, Scott McDougall, Stephen G. Evans, W. Andy Take

**Affiliations:** 1 Department of Earth, Ocean and Atmospheric Sciences, The University of British Columbia, Vancouver, Canada; 2 WSP, Lakewood, CO USA; 3 Knight Piésold, Vancouver, BC Canada; 4 Klohn Crippen Berger, Toronto, ON Canada; 5 Red Earth Engineering, Brisbane, Australia; 6 Department of Earth and Environmental Sciences, University of Waterloo, Waterloo, ON Canada; 7 Department of Civil Engineering, Queen’s University, Kingston, ON Canada

**Keywords:** Tailings dam breach analysis (TDBA), Numerical modelling, Runout analysis, Uncertainty analysis, Sensitivity analysis, FOSM, HEC-RAS 2D

## Abstract

Tailings dam breaches (TDBs) and subsequent flows can pose significant risk to public safety, the environment, and the economy. Numerical runout models are used to simulate potential tailings flows and understand their downstream impacts. Due to the complex nature of the breach-runout processes, the mobility and downstream impacts of these types of failures are highly uncertain. We applied the first-order second-moment (FOSM) methodology to a database of 11 back-analyzed historical tailings flows to evaluate uncertainties in TDB runout modelling and conducted a sensitivity analysis to identify key factors contributing to the variability of the HEC-RAS model output, including at different locations along the runout path. The results indicate that prioritizing resources toward advancements in estimating the values of primary contributors to the sensitivity of the selected model outputs is necessary for more reliable model results. We found that the total released volume is among the top contributors to the sensitivity of modelled inundation area and maximum flow depth, while surface roughness is among the top contributors to the sensitivity of modelled maximum flow velocity and flow front arrival time. However, the primary contributors to the sensitivity of the model outputs varied depending on the case study; therefore, the selection of appropriate rheological models and consideration of site-specific conditions are crucial for accurate predictions. The study proposes and demonstrates the FOSM methodology as an approximate probabilistic approach to model-based tailings flow runout prediction, which can help improve the accuracy of risk assessments and emergency response plans.

**Supplementary Information:**

The online version contains supplementary material available at 10.1007/s10230-024-00970-w.

## Abstract (zh)

尾矿坝溃坝 (TDBs)及后续流量对公共安全、环境和经济造成重大威胁。用数值流动模型模拟潜在尾矿流并了解其下游的影响。由于溃坝流动过程的复杂性, 这类故障的流动性和下游影响是极不确定的。我们将一次二阶矩(FOSM)方法应用到包含11个经过回溯分析的历史尾矿流数据库, 以此来评估TDB流动模型的不确定性, 并进行敏感性分析, 以确定HEC-RAS模型输出 (包括流动路径的不同位置) 变化的关键因素。结果表明, 为了获得更可靠的模型结果, 有必要优先将资源用于估算对所选模型输出的敏感性的贡献值。我们发现总释放量对模拟淹没面积和最大水流深度的敏感性的贡献值最大, 而表面粗糙度对影响模拟水流最大流速和峰值到达时间的敏感性的贡献值最大。然而对模型输出的敏感性的主要贡献因案例研究而异; 因此选择合适的流动模型并考虑具体地点的条件是准确预测的关键。该研究提出并论证了FOSM方法作为基于模型的尾矿流流动预测的近似概率方法, 有助于提高风险评估和应急计划的准确性。

## Abstract (de)

Tailings-Dammbrüche (TDBs) und nachfolgende Abflüsse können ein erhebliches Risiko für die öffentliche Sicherheit, die Umwelt und die Wirtschaft darstellen. Numerische Abflussmodelle werden verwendet, um potenzielle Verlagerungen der Tailings zu simulieren und ihre Auswirkungen auf stromabwärts gelegene Bereiche zu verstehen. Aufgrund der Komplexität der Abflussprozesse sind die Mobilität und die stromabwärts gerichteten Auswirkungen solcher Dammbrüche mit großer Unsicherheit verbunden. In der vorliegenden Studie wurde die FOSM-Methode (First-order-second-moment) auf eine Datenbank mit 11 historischen Tailings-Abflüssen angewendet, um die Unsicherheiten bei der Modellierung des TDB-Auslaufs zu bewerten, und eine Sensitivitätsanalyse durchgeführt, um Schlüsselfaktoren für die Variabilität der HEC-RAS-Modellausgabe zu identifizieren – auch an verschiedenen Stellen entlang des Fließpfades. Die Ergebnisse deuten darauf hin, dass eine Priorisierung der Ressourcen auf die Verbesserung der Schätzung der Werte der wichtigsten Faktoren, die zur Empfindlichkeit der ausgewählten Modellergebnisse beitragen, für zuverlässigere Modellergebnisse notwendig ist. Dabei zeigte sich, dass das freigesetzte Gesamtvolumen mit am stärksten zur Sensitivität der modellierten Überschwemmungsfläche und der maximalen Fließtiefe beiträgt, während die Oberflächenrauhigkeit mit am stärksten zur Sensitivität der modellierten maximalen Fließgeschwindigkeit und der Ankunftszeit der Fließfront beiträgt. Die wichtigsten Faktoren für die Sensitivität der Modellergebnisse können jedoch je nach Fallstudie variieren; folglich sind die Auswahl geeigneter rheologischer Modelle und die Berücksichtigung standortspezifischer Bedingungen entscheidend für genaue Vorhersagen. Die Anwendung der FOSM-Methode als approximativer probabilistischer Ansatz für die modellgestützte Vorhersage des Auslaufens von Tailings zeigt, dass diese dazu beitragen kann, die Genauigkeit von Risikobewertungen und Notfallplänen zu verbessern.

## Resumen

Las roturas de presas de relaves (TDBs) y los flujos subsiguientes pueden suponer un riesgo significativo para la seguridad pública, el medio ambiente y la economía. Los modelos numéricos de desbordamiento se utilizan para simular posibles flujos de relaves y comprender su impacto aguas abajo. Debido a la naturaleza compleja de los procesos de rotura-desbordamiento, la movilidad y los impactos aguas abajo de este tipo de fallos tienen mucha incertidumbre. Se aplicó la metodología del segundo-momento de primer-orden (FOSM) a una base de datos de 11 flujos históricos de relaves analizados retrospectivamente para evaluar las incertidumbres en la modelización del desbordamiento de TDB y se realizó un análisis de sensibilidad para identificar los factores clave que contribuyen a la variabilidad de los resultados del modelo HEC-RAS, incluso en diferentes ubicaciones a lo largo de la trayectoria de fuga. Los resultados indican que es necesario priorizar los recursos hacia avances en la estimación de los valores de los principales contribuyentes a la sensibilidad de los resultados del modelo seleccionado para obtener resultados más fiables del modelo. El volumen total liberado se encuentra entre los principales contribuyentes a la sensibilidad del área de inundación modelizada y la profundidad máxima del flujo, mientras que la rugosidad de la superficie se encuentra entre los principales contribuyentes a la sensibilidad de la velocidad máxima del flujo modelizado y el tiempo de llegada del frente de flujo. Sin embargo, los principales factores que contribuyen a la sensibilidad de los resultados del modelo varían dependiendo del caso de estudio; por lo tanto, la selección de modelos reológicos apropiados y la consideración de las condiciones específicas del emplazamiento son cruciales para obtener predicciones precisas. El estudio propone y muestra la metodología FOSM como un enfoque probabilístico aproximado para la predicción de la extensión de flujos de relaves basada en modelos, que puede ayudar a mejorar la precisión de las evaluaciones de riesgos y los planes de respuesta a emergencias.

## Introduction

### Preamble

Tailings dam breaches (TDBs) and subsequent downstream tailings flows can pose significant risk to public safety, the environment, and the economy (Blight 2009; Ghahramani et al. 2020; Rana et al. 2021a; Santamarina et al. 2019). Runout models have been used to simulate the behaviour and characteristics of potential tailings flows, including inundation area, runout distance, flow velocity, flow depth, and arrival time (Ghahramani et al. 2022; Martin et al. 2019; Pirulli et al. 2017). Researchers use TDB runout modelling to understand the complex physical mechanisms and the downstream impacts of tailings flows in diverse terrains, whereas mine owners and industry consultants rely on the results of TDB analyses (TDBAs) to assign consequence classifications and develop emergency response plans (Canadian Dam Association (CDA) 2021).

A recent benchmarking study by Ghahramani et al. (2022), involving four numerical models commonly used in TDBAs, indicated a high level of uncertainty in model inputs. Some of these uncertainties were attributed to incomplete site-specific observational data and laboratory and in-situ measurements, the resulting challenges associated with selecting proper input parameters (e.g. the estimation of released volume/hydrograph and the selection of rheological models and their associated parameters), and the subjectivity in the model calibration process. The study highlighted the need for additional back-analysis of historical tailings flows to better understand and quantify the sensitivities of output variables in modelling results, and the importance of developing a systematic probabilistic approach for runout analysis in TDBA practice (Ghahramani et al. 2022). The CDA (2021) TDBA guidelines also list additional sources of uncertainties in topographic data quality, failure modes, and triggering factors.

High levels of uncertainty in input variables (e.g. total released volume, rheological parameters, surface roughness, breach parameters) can in turn lead to high uncertainty in output variables (e.g. runout distance, inundation area, flow velocity). The uncertainty in model outputs is quantified by studying the distribution of possible outcomes with respect to the uncertainty in input parameters. This type of uncertainty analysis is useful when evaluating the reliability and accuracy of model results and has been a practice for decades in various engineering activities, such as structural, geotechnical, hydraulic, aerospace, and manufacturing processes (e.g. Baecher and Christian 2005; Burges and Lettenmaier 1975).

**Fig. 1:**
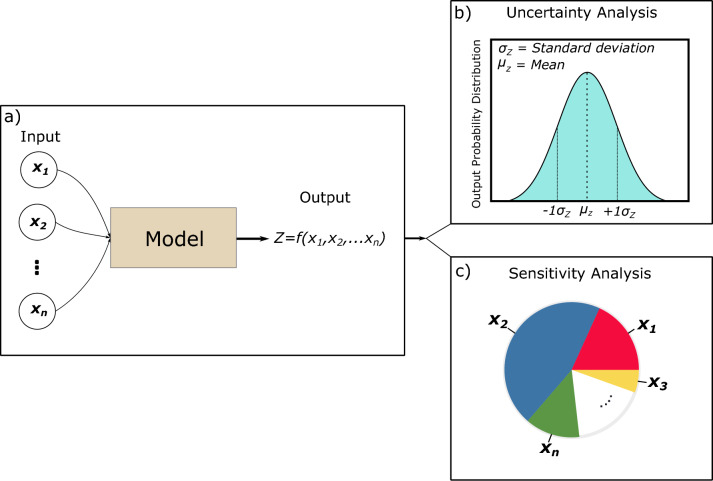
Framework illustration of numerical model uncertainty and sensitivity analyses and distinctions between them. Box (**a**) displays a numerical model with its input and output variables. Box (**b**) illustrates the results of uncertainty analysis, which provides the distribution of possible outcomes with respect to the uncertainty in input parameters while box c) illustrates the results of sensitivity analysis, which identifies the dominant controls of the uncertainty in modelling results

Identifying the dominant controls of the uncertainty in modelling results can help determine which inputs require further consideration and/or higher investments in time/budget. One method to achieve this is a sensitivity analysis, the aim of which is to investigate how changes in input variables affect the output results (Borgonovo and Plischke 2016; Razavi et al. 2021). Uncertainty and sensitivity analyses are related, but have different meanings and purposes. Figure 1 illustrates the concept of uncertainty and sensitivity measures and the distinction between them. Together, uncertainty and sensitivity analyses can help the modeller understand, enhance, and communicate the quality and reliability of the model outcomes to support well-informed decision-making.

To quantify uncertainty, probabilistic methods such as first-order second-moment (FOSM) (Baecher and Christian 2005; Lee and Mosalam 2005; Llano-Serna et al. 2018; Kim et al. 2020; Nadim 2007) and Monte Carlo simulations (MCS) (Kleijnen 1995; Razavi et al. 2021; Tonkin and Doherty 2009) have become popular. The FOSM method has been shown to be a computationally efficient tool in different engineering applications where more computationally expensive methods, such as MCS, are not possible (e.g. Kunstmann and Kinzelbach 2000; Kunstmann et al. 2002; Nadim 2007; Wang and Hsu 2009). This method approximates the mean and variance of a model output variable of interest as a function of the mean and variance of the input factors and their correlations (Baecher and Christian 2005). An advantage of the FOSM method is that it can provide the uncertainties of an output variable from each input variable separately and/or by considering all input variables together (Kim et al. 2020). This method has been used for uncertainty quantification in water quality modelling and groundwater modelling (Dettinger and Wilson 1981; Kunstmann and Kinzelbach 2000; Kunstmann et al. 2002; Wang and Hsu 2009), for the analysis of the probability of geotechnical failure and potential consequences (Baecher and Christian 2005; Kim et al. 2020; Nadim 2007), and to investigate the sensitivity of the seismic demand of a structure to potential future earthquakes (Lee and Mosalam 2005). This track record of success in related problems made the FOSM method a promising candidate to capture the uncertainty in TDB runout modelling in this study.

### Scope and Objectives

Various hydraulic modelling and landslide runout modelling tools are available for TDBAs (Canadian Dam Association (CDA) 2021; Ghahramani et al. 2022; McDougall 2017). The entire breach-runout process in a tailings dam is complex and strongly dependent on site-specific conditions, and the physical mechanisms of tailings flows remain poorly understood. As such, simplifications are made at almost every stage of the model development, from the mathematical differential equations to the initial and boundary conditions. In addition, there is uncertainty associated with the estimation of the model inputs. As a result, there are different sources of uncertainty associated with numerical models (Ghahramani et al. 2022; Martin et al. 2022; Pirulli et al. 2017). To the best of our knowledge, only one recent study (Melo and Eleutério 2023) has investigated the sensitivity of tailings dam breach inundation mapping to rheological parameters through a probabilistic approach and those authors highlighted the lack of research on probabilistic approaches, particularly for TDBAs.

To address this gap, we used a database of 11 back-analyzed tailings flow cases to assess the uncertainties in TDB runout modelling using the FOSM method. The Hydrologic Engineering Centre’s River Analysis System (HEC-RAS) numerical model, developed as a publicly accessible tool by the U.S. Army Corps of Engineers, was used for the back-analysis (Adria 2022; Brunner 2020). The main objectives of this study were to: (1) identify the primary contributors to the sensitivity of key model outputs (inundation area, maximum flow velocity, maximum flow depth, and front flow arrival time) among the selected input variables (total released volume, yield stress, viscosity, surface roughness, breach width, and breach formation time), (2) study the variation of sensitivity estimates along the flow runout path, and (3) investigate the applicability of the FOSM method for probabilistic runout modelling in prediction applications.

## Methodology

### The FOSM Method

The FOSM approach is a numerical probabilistic method in which the mean and variance of the model output variables can be estimated by the first-order approximation of a Taylor series expansion, using the mean and variance of the input variables (Baecher and Christian 2005; Nadim 2007). If the number of uncertain input variables is *n*, this method requires either evaluating $n$ partial derivatives of the performance function or performing a numerical approximation using evaluations at $2n+1$ points. We used the latter approach in this study. For an output function $Z={f}_{\left({X}_{1},{X}_{2}, \dots {X}_{n}\right)}$*,* in which ${X}_{1},{X}_{2}, \dots {X}_{n}$ are random variables, using the first-order approximation, the mean, *μ*_*z*_, and variance, *σ*_*z*_^2^, of the function *Z,* become:

1 $${\mu }_{Z}\approx {f(\mu }_{{X}_{i}})={f(\mu }_{{X}_{1}},{\mu }_{{X}_{2}}, \dots {\mu }_{{X}_{n}})$$

2 $${\sigma }_{Z}^{2}\approx \sum_{i=1}^{n}{\sigma }_{{X}_{i}}^{2}{(\frac{\partial f}{\partial {x}_{i}})}^{2}+{\sum }_{i=1}^{n}{\sum }_{j\ne i}^{n}\frac{\partial f}{\partial {x}_{i}}\frac{\partial f}{\partial {x}_{j}} COV({X}_{i},{X}_{j})$$

where: ${\mu }_{{X}_{i}}$ and ${\sigma }_{{X}_{i}}$ ^2^ are the means and the variances of model inputs for $i=1, 2,\dots ,n$, $n$ is the number of inputs, and $COV({X}_{i},{X}_{j})$ is the covariance between input variables ${X}_{i} {\text{and}} {X}_{j}$. If it is assumed that the variables are uncorrelated, the second term on the right side of Eq. 2 vanishes (Baecher and Christian 2005; Nadim 2007).

Although FOSM is a linearization technique, it can be applied to models with non-linear output functions. The FOSM method linearizes the non-linear output function by approximating it as a Taylor series expansion around the mean values of the input variables. Therefore, it assumes that the output can be locally approximated as a linear function near the mean values of the input variables (Lee and Mosalam 2005).

### Output Variables

The output variables of TDB runout modelling represent the simulated characteristics of the tailings flow downstream of the breach. For a FOSM analysis, four main outputs are studied: inundation area, maximum flow velocity, maximum flow depth, and flow front arrival time. The output values of the last three variables are measured at 50% of the observed Zone 1 runout distance, which is defined as “the extent of the main solid tailings deposit, which is characterized by remotely visible or field-confirmed sedimentation, above typical bankfull elevations if extending into downstream river channels” (Ghahramani et al. 2020).

### Input Variable Statistics

In this study, the following six input variables were selected: total released volume, yield stress, viscosity, surface roughness, breach width (considering a trapezoidal breach shape), and breach formation time. Detailed definitions of breach geometry and breach formation time are provided in Wahl (1998) and Froehlich (2008). The conventions are adopted from water-retaining dam breach practice, as they were found to be generally suitable for tailings dam breaches by Adria et al. (2023a). Breach formation time is only used for erosional breach case studies that typically involve overtopping or piping/seepage with a relatively large volume of supernatant pond and a long breach duration. In reality, some of these inputs might be correlated (e.g. a wider breach can release more tailings and higher yield stress values are typically associated with higher viscosity values). However, in the HEC-RAS numerical model, the six selected inputs are formulated independently and are manually assigned, and therefore are not correlated. In other words, adjusting the breach width value in HEC-RAS does not affect the value for the outflow volume, or adjusting the yield stress value does not affect the viscosity value. Input variables that can be correlated to other inputs in the HEC-RAS model, such as solid concentration, were not considered in this study. For example, as part of the quadratic rheology within the numerical model, yield stress and viscosity are both a function of solid concentration. The best-fit (calibrated) input values that are estimated in the case study back analyses (described in the “Tailings flow back-analyses” section) are set to be the mean of the model inputs (${\mu }_{{X}_{i}})$ and their output results are represented by the mean values (${\mu }_{Z})$ of the model outputs. Other programs or modelling tools used in TDBAs may treat input values differently than HEC-RAS.

There are two ways to estimate the variance, ${\sigma }_{Z}^{2}$, of the output function *Z*: i) if the function ${f}_{\left({X}_{1},{X}_{2}, \dots {X}_{n}\right)}$ is tractable, the function can be differentiated to give a closed-form expression for the variance of ${f}_{\left({X}_{1},{X}_{2}, \dots {X}_{n}\right)}$ or ii) more commonly, it is not possible to differentiate the function directly; therefore, the partial derivatives must be obtained through numerical approximation approaches (Baecher and Christian 2005). In this application, since the form of function *Z* is unknown, the second approach is used to approximate the partial derivatives with the central differences method. To find the partial derivative for each best-fit input variable, the best-fit input value is increased and decreased by a small increment (± 10% was used in this study), while the rest of the variables are kept constant. The differences between the resulting output values are then calculated and divided by the differences between the increased and decreased input values. This can be represented mathematically as follows:

3 $$\frac{\delta {f(\mu }_{{X}_{i}})}{\delta {x}_{i}}\approx \frac{1}{{2\varepsilon }_{i}}\left\{{f}_{\left({\mu }_{{X}_{1}},{\mu }_{{X}_{2}},\dots ,{\mu }_{{X}_{i}}+{\varepsilon }_{i},\dots ,{\mu }_{{X}_{n}}\right)}\right.-\left.{f}_{\left({\mu }_{{X}_{1}},{\mu }_{{X}_{2}},\dots ,{\mu }_{{X}_{i}}-{\varepsilon }_{i},\dots ,{\mu }_{{X}_{n}}\right)}\right\}$$

where ${\varepsilon }_{i}$ is ± 10% of the best-fit value for the particular input.

To compute Eq. 2, an estimate of the variance of the model inputs is also needed. To achieve this, the standard deviation and the mean values of selected variables were estimated statistically using data from available databases. For the total released volume and breach width, data from 41 TDB cases and 36 TDB cases, respectively, were collected from Rana et al. (2021b). Since the total released volume is a portion of the total impoundment volume, the ratio of the total released volume to the total impoundment volume was used to estimate of the mean and standard deviation. For the breach width statistics, the top breach width data from Rana et al. (2021b) were used, and for the FOSM analysis, the side slopes were kept constant. For the surface roughness, 74 data points from Chow (1959) were used. For the breach formation time statistics, 27 water retaining dam failures were compiled from Wahl (1998) and Wahl (2014). Ghahramani et al. (2022) and Adria (2022) showed that the numerical rheological parameter values do not necessarily correspond with the measured rheological parameter values. However, in the absence of sufficient calibrated yield stress and viscosity data from the numerical back-analysis of historical cases, a tailings rheology database from Martin et al. (2022) was used as a first order approximation for estimating the mean and standard deviation of rheological parameters. Using the rheology database, the yield stress and viscosity data were classified with respect to the volumetric solid concentration ranges and their means and standard deviations were estimated for each range. Then, the coefficients of variation (CoV) of input variables were calculated as the ratio of their standard deviation to their mean values. Tables 1, 2, 3 present the input variables with their estimated CoV values. A greater CoV indicates greater dispersion around the mean value. Finally, the standard deviation of a model input can be estimated for each case study individually, by multiplying the best-fit value (mean) of the model input and estimated CoVs.

**Table 1:** Coefficient of variation (CoV) values for the selected input variables

Input parameter	Mean	Standard deviation	CoV	Sample size	References
Total released volume^a^ (%)	36	25	0.7	41	Rana et al. (2021b)
Roughness (s/m^1/3^)	0.06	0.05	0.8	74	Chow (1959)
Yield stress (Pa)	36–297	74–127	0.4–2.1	See Table 2	Martin et al. (2022)
Viscosity (Pas)	0.27–1.6	0.1–2.4	0.4–1.5	See Table 3	Martin et al. (2022)
Breach width (m)	218	269	1.2	36	Rana et al. (2021b)
Breach formation time^b^ (m/h)	31	29	0.9	27	Wahl 1998 and Wahl 2014

^a^The ratio of the total released volume to the total impoundment volume

^b^The breach formation time is normalized by the breach height similar to the mean erosion rate in Walder and O’Connor (1997)

**Table 2:** Coefficient of variation (CoV) values for the yield stress with respect to volumetric solid concentration (*C*_v_)

Parameter	*C*_v_ range (%)	Sample size	CoV
Yield stress	10–19.9	21	0.9
	20–29.9	73	1.6
	30–39.9	121	2.1
	40–49.9	59	1.6
	50–59.9	49	1.5
	60–69.9	10	0.4

Data extracted from the database in Martin et al. (2022)

**Table 3:** Coefficient of variation (CoV) values for the viscosity with respect to volumetric solid concentration (*C*_v_)

Parameter	*C*_v_ range (%)	Sample size	CoV
Viscosity	10–19.9	7	0.6
	20–29.9	31	0.9
	30–39.9	59	1.5
	40–49.9	17	1.2
	50–59.9	5	0.4
	60–69.9	–	–

Data extracted from the database in Martin et al. (2022)

### Sensitivity and Uncertainty Estimates of Model Outputs

A type of sensitivity analysis can be carried out using differentiation-based methods (Borgonovo and Plischke 2016). The FOSM methodology enables estimation of the gradient of the output variables with respect to the input variables, due to small local changes in model inputs. Therefore, the partial derivatives in Eq. 2 become natural sensitivity estimates. Since the partial derivative of each input has different units from one another, we use the equation provided by Borgonovo and Plischke (2016), in which the sensitivity measures (*D*_*i*_) are normalized and can be ranked (Eq. 4). The result of this sensitivity analysis can be used to identify the primary contributors to the uncertainty in model outputs. The uncertainty measure (CoV_Z_) of each output is estimated as the ratio of the standard deviation to the mean value, which is called the coefficient of variation of the output variable (Eq. 5).

4 $${D}_{i}= \frac{\left|\frac{\partial {f}_{{(\mu }_{X})}}{\partial {X}_{i}} d{X}_{i}\right|}{\left|\sum_{j=1}^{n}\frac{\partial {f}_{{(\mu }_{X})}}{\partial {X}_{j}} d{X}_{j}\right|}$$

where ${X}_{i}$ is the input variable and n is the number of inputs. This fraction quantifies how the resulting output value changes with a particular input variable relative to the total change in the output variable.

5 $${CoV}_{Z}= \frac{{\sigma }_{Z}}{{\mu }_{Z}}$$

where ${\sigma }_{Z}$ and ${\mu }_{Z}$ are the standard deviation and mean of model output, respectively. The estimated standard deviation and mean of each output are obtained from the FOSM results.

In this study, the sensitivity analysis was divided into two parts. For the first part, the 50% runout distance of Zone 1 was selected for all local model sensitivity estimates as a consistent relative location to compare all events for maximum flow velocity, maximum flow depth, and flow front arrival time. For the second part, the variation of sensitivity estimates was investigated at 10%, 25%, 50%, 75%, and 90% of the Zone 1 runout distance.

### Tailings Flow Back-Analysis

#### HEC-RAS 2D

HEC-RAS is an open-access software package that was originally developed by the U.S. Army Corps of Engineers (USACE) for water resource engineering and open-channel hydraulic analysis (Adria 2022; Brunner 2020; Gibson et al. 2021, 2022). HEC-RAS 2D is a depth-integrated two-dimensional model that uses the finite volume numerical method. It is capable of dam breach-runout modelling, erosion and sediment transport simulations, and water quality analyses (Brunner 2020). Version 6.1 of HEC-RAS 2D (the most current version at the time of this work) was used in this work due to its popular application in dam breach-runout modelling and flood risk management studies, and its demonstrated capability of modelling both Newtonian and non-Newtonian flow types (Adria 2022; Brunner 2020; Gibson et al. 2021). There are four selectable options of rheological models for non-Newtonian flow simulations: Bingham, Quadratic, Herschel-Bulkley, and Voellmy.

#### Back-Analyzed Case Studies

The back-analyses of the 11 historical cases that we used as baseline models in the FOSM analysis are detailed in Adria (2022). The original database is provided in an open-access data repository hosted at Borealis (Adria et al. 2023b). These cases are selected based on the availability of information on the pre-and post-failure site and tailings characteristics. Classifying the case studies based on the type of breach process (CDA 2021), there are three erosional breach and eight non-erosional breach case studies (Table 4). Overtopping and piping/seepage type of failure mechanisms commonly involve an erosional breach process with a relatively large volume of supernatant pond and a long breach duration, from several minutes to hours. The non-erosional breach processes involve near-instantaneous collapses and have characteristics other than the erosional breaches mentioned above (CDA 2021). In Table 4, the Tapo Canyon Event 1 refers to the viscous section of the Tapo Canyon tailings flow and the Cadia Event 2 refers to the secondary liquefaction event, which occurred on March 11, 2018; more details are provided in Adria (2022).

**Table 4:** Database of 11 back-analyzed tailings dam breaches (TDBs)

ID	TDB case	Country	Year	Breach process
1	Stava	Italy	1985	Non-erosional
2	Tapo Canyon Event 1	USA	1994	Non-erosional
3	Merriespruit (Harmony)	South Africa	1994	Non-erosional
4	Aznalcóllar (Los Frailes)	Spain	1998	Erosional
5	Ajka (Kolontar)	Hungary	2010	Erosional
6	Kayakari	Japan	2011	Non-erosional
7	Mount Polley	Canada	2014	Erosional
8	Fundão	Brazil	2015	Non-erosional
9	Tonglvshan	China	2017	Non-erosional
10	Cadia Event 2	Australia	2018	Non-erosional
11	Feijão	Brazil	2019	Non-erosional

The topographic data used in the models consisted of a mix of publicly available and commercial sources, with additional manual modifications as needed. The breach characteristics and outflow volumes for each event were previously compiled in Adria (2022), Ghahramani et al. (2020), and Rana et al. (2021a). The yield stress and viscosity in the quadratic rheological model were calibrated in two steps. First, the modelled inundation area was compared to the observed inundation area as mapped by Ghahramani et al. (2020) and Rana et al. (2021a) using a quantitative method developed by Heiser et al. (2017). The modelled results were then compared to available observations of arrival time and runout depth within the inundation area to further refine the calibrated yield stress and viscosity. The quadratic rheological model as implemented in HEC-RAS also uses a third term that relates shear stress to strain-rate squared, to simulate dispersive effects. The coefficient for the dispersive term is calculated with a combination of theoretical, empirical, and measurable sediment characteristics (e.g. particle diameter). Only the particle diameter was varied in Adria (2022) based on available data for each event. The calculated dispersive coefficients between all events ranged from 1.1 × 10^–5^ to 6.1 × 10^–2^, which aligns with the findings of Julien and Lan (1991). The surface roughness was defined with spatially varied Manning’s *n* values based on the land cover observed on satellite/aerial imagery, as well as guidance in Arcement and Schneider (1989) and Janssen (2016), but it was not adjusted as part of the calibration process. The best-fit model inputs and outputs are presented in Table 5.

**Table 5:** List of the best-fit input values and the best-fit modelled outputs from the back-analyses of 11 TDBs

ID	TRV (mm^3^)	YS (Pa)	V (Pa s)	R	TBW (m)	Inundation area (m^2^)	Max velocity^a^ (m/s)	Max depth^a^ (m)	Arrival time^a^ (s)
1	0.1852	3.2	1.8	0.04	220	522,608	18.23	6.25	202
2	0.0275	4000	4	0.06	145	19,136	6.57	3.84	18.8
3	0.615	200	4	0.08	150	1,021,109	2.09	1.97	249
4	6.75	2.5	0.4	0.055	84	15,419,072	1.26	3.83	12,468
5	1.2	3.2	3.2	0.04	60	7,050,620	0.82	0.58	21,010
6	0.041	250	15	0.03	50	108,094	5.98	1.93	147
7	25	2	0.02	0.04	262	2,987,242	6.16	11.29	1980
8	32.2	3	0.05	0.07	650	10,216,937	2.49	14.16	6,460
9	0.5	250	2.5	0.04	260	278,761	6.27	3.18	34.6
10	0.16	1780	562	0.02	30	103,077	5.20	4.23	36
11	9.65	400	25	0.08	560	3,700,751	5.98	9.41	679

Numbers represent case IDs given in Table 4

*TRV* reported total released volume, *YS* best-fit yield stress, *V* best-fit viscosity, *R* estimated surface roughness, *TBW* reported top breach width

^a^At the 50% observed runout distance

In the FOSM analysis, as described earlier, the number of evaluation points is 2*n* + 1 (where *n* is the number of model inputs) when the numerical approximation method is used. Considering the five and six inputs for non-erosional and erosional breach case studies, respectively, each non-erosional breach case study was run 11 times, and each erosional beach case study was run 13 times. Therefore, in total, there were 127 evaluation points for all 11 case studies. Refer to Supplementary Appendix A for all of the performed runs.

To investigate the variation of sensitivity estimates along the flow runout distance, three case studies were selected (1985 Stava, 1998 Aznalcóllar, and 2019 Feijão). These cases were selected because they have different released volumes, breach processes, and topographic conditions, and therefore the magnitude and runout path of these three cases represent diverse morphological environments (see Tables 4 and 5).

### Probability Distribution Approximation for Prediction

The FOSM method provides estimates of the mean and standard deviation of the model outputs, which can be used to make probabilistic forward predictions. However, since the probability distributions of the inundation area, maximum flow velocity, maximum flow depth and frontal arrival time are unknown and are not obtained directly through the FOSM method, assumptions must be made. Normal and log-normal approximations are typically used in geotechnical problems (Kim et al. 2020; Nadim 2007). The log-normal approximation is also considered reasonable in this application, as a first approximation. It is used instead of the normal distribution because the model input parameters cannot be negative.

The Merriespruit case was used in this study to demonstrate the application of the FOSM method for predicting the probability of model outputs. Following Aaron et al. (2022), we excluded any site-specific information about the breach geometry, rheological parameters and observational data for the numerical modelling. The Merriespruit case was also excluded from the TDB calibration dataset for the purposes of this demonstration. However, the total released volume was not changed so that the observed and simulated results could be roughly compared.

A detailed description of the Merriespruit TDB event was provided by Fourie and Papageorgiou (2001) and Wagener (1997). The estimated total released volume was 0.615 M m^3^. For the purpose of demonstrating the probabilistic method, we adopted a strategy of making reasonable model and parameter selections that an experienced TDB practitioner might make. A trapezoidal breach shape with side slopes of 1 V:1H was used. Assuming the breach height was equal to the dam height (31 m), and using an average breach width to breach height ratio of 7 based on non-erosional breach data in Adria (2022), the average breach width was estimated to be 217 m. The topographic data source is the Airbus WorldDEM™ DTM with a 12 m resolution. A constant surface roughness (n) of 0.08 was used throughout the runout path to account for both the suburban and wetland areas that are observed in satellite imagery and aerial photographs. The Quadratic rheological model was selected for this analysis. Since there is a lack of back-analyzed historical case studies similar to Merriespruit, which could otherwise inform rheological parameter selection, the yield stress and viscosity in the present study were estimated by fitting exponential curves to the yield stress and viscosity data provided in Martin et al. (2022). Considering the volumetric solid content of 50% for Merriespruit, the yield stress and viscosity were estimated as 63 Pa and 0.8 Pa s, respectively.

## Results

### Sensitivity and Uncertainty Estimates

**Fig. 2:**
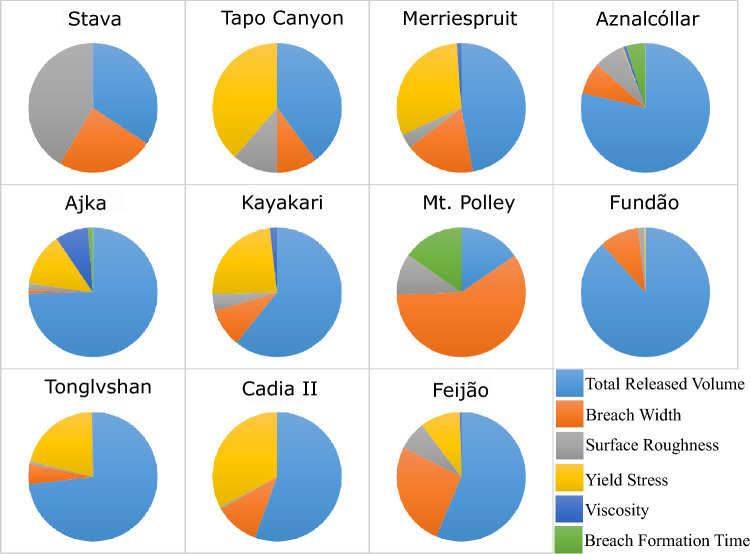
Sensitivity of modelled inundation area with respect to the selected inputs

**Fig. 3:**
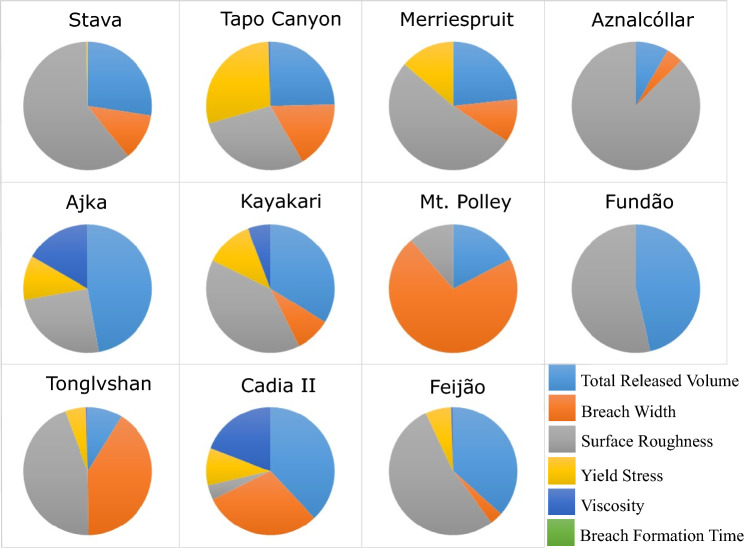
Sensitivity of modelled maximum flow velocity at 50% runout with respect to the selected inputs

**Fig. 4:**
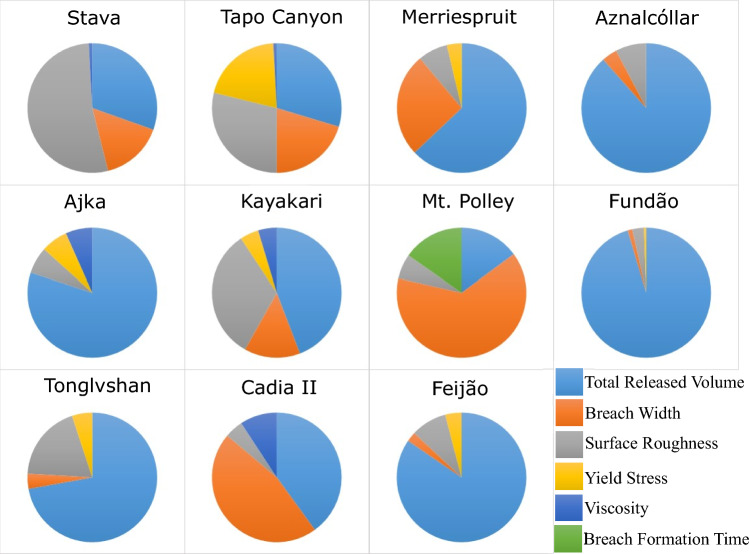
Sensitivity of modelled maximum flow depth at 50% runout with respect to the selected inputs

**Fig. 5:**
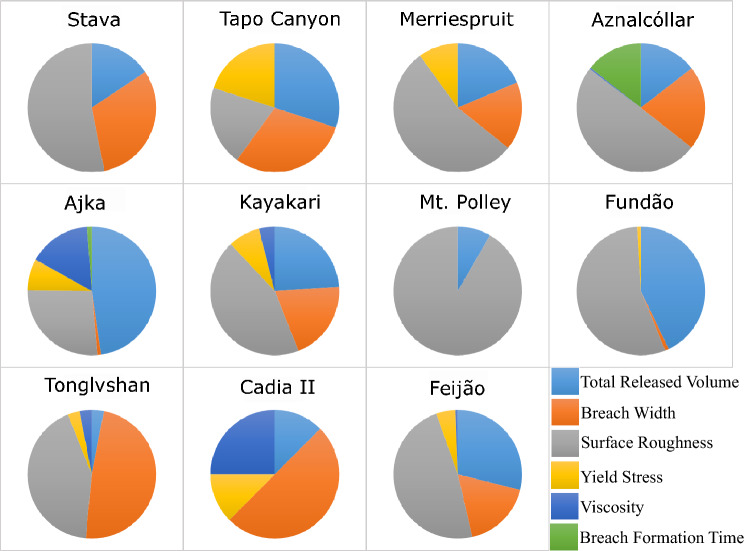
Sensitivity of modelled flow front arrival time at 50% runout with respect to the selected inputs

Figures 2, 3, 4 and 5 illustrate the sensitivity of the modelled inundation area, maximum flow velocity at 50% runout, maximum flow depth at 50% runout, and flow front arrival time at 50% runout, respectively, for each case study. Figure 2 indicates that the inundation area was most sensitive to total released volume in 9 out of 11 cases, with the exceptions of Stava and Mt. Polley. Stava exhibited the greatest sensitivity to surface roughness, while Mt. Polley exhibited the greatest sensitivity to breach width. Yield stress was one of the top two contributors to the sensitivity of inundation area for more than half of the cases (6 out of 11) (Fig. 2).

Figures 3 and 4 indicate that the maximum flow velocity and maximum flow depth at 50% runout were most sensitive to surface roughness and total released volume, respectively, in 8 out of 11 cases. For flow velocity, the exceptions are Cadia, Mt. Polley, and Ajka; Cadia and Ajka exhibit the highest sensitivity to total released volume, while Mt. Polley exhibits the highest sensitivity to breach width (Fig. 3). For flow depth, the exceptions were Stava, Mt. Polley, and Cadia; Mt. Polley and Cadia exhibit the highest sensitivity to breach width, while Stava exhibits the highest sensitivity to surface roughness (Fig. 4).

Figure 5 indicates that the sensitivity results for flow front arrival time display greater variability than the other model outputs. Flow front arrival time is most sensitive to surface roughness in 7 out of 11 cases, with the exceptions of Tapo Canyon, Ajka, Tonglvshan, and Cadia. Tapo Canyon exhibits the highest sensitivity to both total released volume and breach width equally, while Ajka exhibits the highest sensitivity to total released volume, and Tonglvshan and Cadia exhibits the highest sensitivity to breach width.

**Fig. 6:**
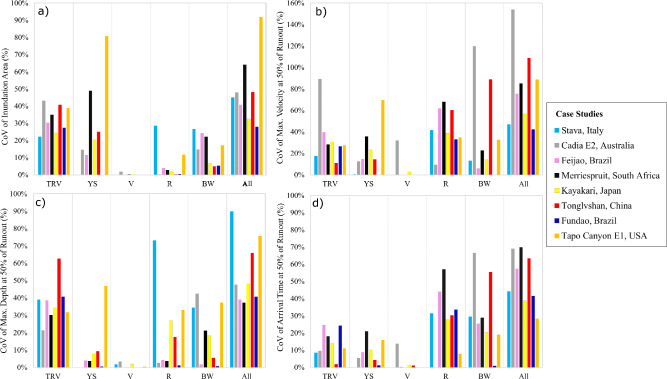
Non-erosional breach case study coefficient of variation (CoV) values for four numerical outputs: **a** inundation area, **b** maximum flow velocity, **c** maximum flow depth, and **d** flow front arrival time at 50% of the observed runout distance. These CoV values are presented with respect to the five selected inputs, namely total released volume (TRV), yield stress (YS), viscosity (V), surface roughness (R), and breach width (BW), both individually and all inputs (All) together. Note the different y-axis range for plot b)

**Fig. 7:**
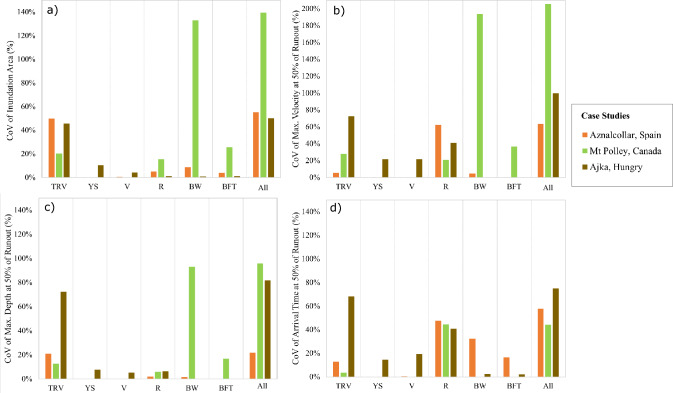
Erosional breach case study coefficient of variation (CoV) values for four numerical outputs: **a** inundation area, **b** maximum flow velocity, **c** maximum flow depth, and **d** flow front arrival time at 50% of the observed runout distance. These CoV values are presented with respect to the six selected inputs, namely total released volume (TRV), yield stress (YS), viscosity (V), surface roughness (R), breach width (BW), and breach formation time (BFT), both individually and all inputs (All) together. Note the different y-axis range for plot b)

Figures 6 and 7 display the uncertainty estimates, CoV (coefficient of variation), values for four numerical outputs: inundation area, maximum flow velocity, maximum flow depth, and flow front arrival time at 50% of the observed runout distance. These values are shown for both non-erosional breach (Fig. 6) and erosional breach (Fig. 7) case studies, considering the selected inputs individually as well as all inputs together (all). The erosional breach case studies include an additional input, breach formation time (BFT), which is not applicable for the non-erosional breach case studies (Fig. 7). The findings suggest that for all case studies, the uncertainties in the inundation area and maximum flow depth with respect to the total released volume exceed 10%, which is typically regarded as a high level of uncertainty in practice. The uncertainties in the maximum flow velocity and flow front arrival time with respect to surface roughness exceed 10% for most of the case studies, specifically 10 out of 11 and 9 out of 11, respectively. Tables containing sensitivity and uncertainty values are presented in Supplementary Appendix B.

### Sensitivity Variation Along Runout Path

**Fig. 8:**
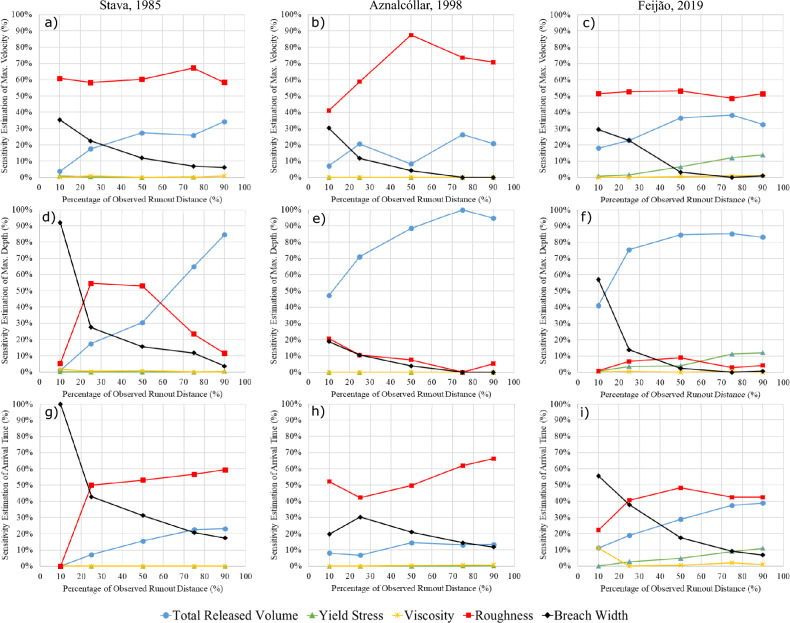
Variation of sensitivity with distance from the breach for modelled maximum flow velocity (**a**, **b**, **c**), maximum flow depth (**d**, **e**, **f**), and frontal arrival time (**g**, **h**, **i**) in three selected case studies

Figure 8 shows the sensitivity of the modelled maximum flow velocity (a, b, c), maximum flow depth (d, e, f) and flow front arrival time (g, h, i) at 10%, 25%, 50%, 75% and 90% of the Zone 1 runout distance for the Stava, Aznalcóllar and Feijão cases. Sensitivity variation is observed along the flow path. In most of the scenarios, the sensitivity to breach width tends to decrease with distance from the breach. The sensitivity to total released volume has an increasing trend in most cases, which appears to plateau in some cases. The sensitivity to surface roughness is largely case-dependent without any discernible common trend. The sensitivity to yield stress tends to increase with distance from the breach in the case of Feijão. Sensitivity values along the runout path are provided in Supplementary Appendix C.

### Demonstration of Probabilistic Prediction Approach

**Fig. 9:**
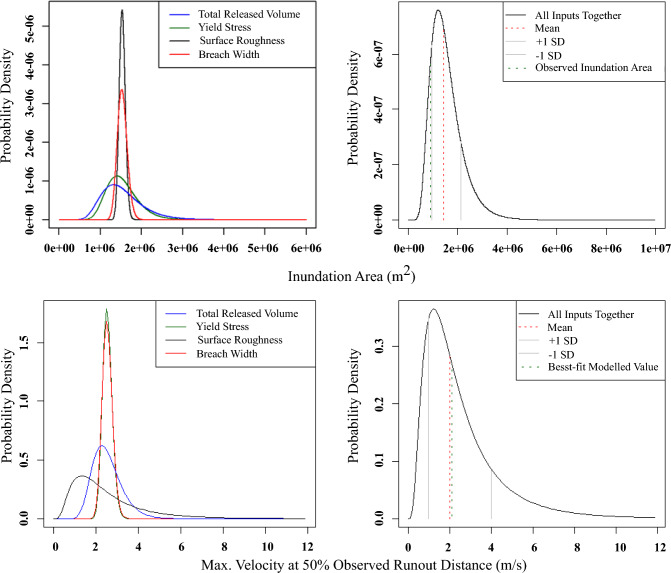
The probability density of the modelled inundation area and maximum flow velocity at 50% of the Zone 1 runout distance are plotted with respect to each input variable (**a** and **c**), as well as considering the total uncertainty of all input variables together (**b** and **d**)

The Merriespruit demonstration case involves modelling the probability distributions of two key parameters: the inundation area and maximum flow velocity at 50% runout distance. These parameters are modelled with respect to each input variable individually as well as considering the total uncertainty of all input variables together (Fig. 9). The mean value of the modelled inundation area is 1.54 km^2^, while the mean value of the modelled maximum flow velocity at 50% runout distance is 2.6 m/s.

Since the model outputs cannot be negative, we used the assumption of log-normal distribution. The observed Zone 1 inundation area for Merriespruit was estimated to be 0.89 km^2^ (Ghahramani et al. 2022), which is slightly outside of one standard deviation from the mean of the probability density curves for the inundation area, as shown in Fig. 9b. Similarly, the best-fit modelled maximum flow velocity at 50% of the runout distance was ≈ 2.1 m/s (Adria 2022), which is within one standard deviation from the mean of the probability density curves for maximum flow velocity, as shown in Fig. 9d.

## Discussion

### Sensitivity Analysis

The results of the sensitivity analysis suggest that modelled inundation area and maximum flow depth are most sensitive to total released volume, whereas modelled maximum flow velocity and flow front arrival time are generally most sensitive to surface roughness. These findings are conceptually consistent with physical observations (Adria 2022; Ghahramani et al. 2020) and agree with past findings that outflow volume is strongly correlated with inundation area and runout distance (e.g. Concha-Larrauri and Lall 2018; Ghahramani et al. 2020; Piciullo et al. 2022; Rico et al. 2008).

With regard to identifying primary contributors to the sensitivity of model outputs, our results indicated similar trends for most of the cases, with a few exceptions for each model output. For Stava, the sensitivity of all four outputs followed a similar pattern, with surface roughness as the primary contributor and total released volume and breach width among the top three contributors to the sensitivity of those model outputs. This may be attributed to the steep travel path at Stava, which is higher than all the other cases in this study.

Mt. Polley is another exception in which the model is highly sensitive to the breach width for the modelled inundation area, maximum flow velocity, and maximum flow depth (Figs. 2, 3, 4). This is likely due to the unique site conditions related to the Mt. Polley failure. The Zone 1 extent of Mt. Polley was truncated by Quesnel Lake 9 km downstream of the tailings facility. Without the presence of an intercepting water body, a hypothetical failure of similar size and composition to Mt. Polley would be expected to travel farther than 9 km. As a result, the 50% runout distance point considered in this study for the Mt. Polley model may actually be more representative of the 5–15% range if the event was not truncated by the lake. From this perspective, the Mt. Polley sensitivity results for all parameters are less exceptional to the other events, as the Stava, Aznalcóllar, and Feijão results are also consistently sensitive to breach width at about 5–15% of their runout distances. Furthermore, for confined events like Mt. Polley, the sensitivity of inundation area to outflow volume is primarily driven by the runout distance, with minor changes in the flow width along the runout path. With the truncated runout distance at Mt. Polley enforcing the same runout distance for all sensitivity scenarios, along with a predominantly channelized flow path, there was physically little room for the inundation area to differ between input variations.

Another consideration for Mount Polley is that the released volume had a relatively low concentration of tailings solids, and therefore could be reasonably approximated as Newtonian rather than non-Newtonian. The effect of low solids concentrations is implicitly included in HEC-RAS by using low values for the yield stress and viscosity. As a result, one could expect the inundation area to have low sensitivity to the low calibrated yield stress and viscosity values for Mt. Polley, which in turn increases the relative sensitivity of the other inputs. This rationale may apply to the Aznalcóllar and Ajka cases as well, which also had relatively low concentrations of tailings solids.

In the case of Cadia, the modelled maximum flow velocity was most sensitive to total released volume, while the modelled maximum flow depth and flow front arrival time were most sensitive to breach width. In general, surface roughness acts as an external resisting force along the flow runout path, and typically, changing the surface roughness affects the modelled flow velocity and arrival time the most. However, this was not the case for Cadia. However, the breach width was one of the primary contributors to the sensitivity of the modelled flow velocity, depth, and flow front arrival time. One possible reason might be the proximity of our measurement to the breach. The Cadia runout distance was ≈ 480 m, which is relatively short, and the sensitivity analysis was done at 50% of the runout distance. Tonglvshan is the only other case that has a similar runout distance to Cadia (≈ 500 m), and breach width was also one of the main contributors to the sensitivity of modelled maximum flow velocity and flow front arrival time in that case. Also, the Manning’s *n* value used for Cadia was relatively low, as expected for barren land (Janssen 2016). The released tailings had a solid concentration of ≈ 63% (Jefferies et al. 2019), and the calibrated yield stress and viscosity for the tailings were among the highest used in Adria (2022), as expected for a material that predominantly consisted of solids. The influence of surface roughness (external flow resistance) should therefore be expected to be less consequential than rheology (internal flow resistance), which is observed for Cadia in Fig. 5. Another possible reason might be related to the selected rheological model. The Quadratic rheology was used for the back-analysis of the Cadia case but considering the high solid concentration of the Cadia tailings, rheological models developed for solid-dominated materials (e.g. Voellmy rheology) might have been more appropriate.

The FOSM results presented in this study pertain specifically to the HEC-RAS numerical model. In Ghahramani et al. (2022), the FOSM analysis revealed that each of the four models (DAN3D, MADflow, FLO-2D, and FLOW-3D) investigated in their study was sensitive to different input parameters. However, the total released volume was identified as one of the top three contributors to the sensitivity of modelled maximum flow velocity and depth at a specific location for all four models. The results of the FOSM analysis conducted in this study are consistent with those findings.

### Sensitivity Variation

The results in the “Sensitivity Variation Along Runout Path" section indicate that the sensitivity of model outputs to model inputs varies at different locations along the runout path. This is consistent with a parallel complementary study on the analogous problem of landslide runout that recognized sensitivity variation over the extent of a landslide runout model (Mitchell et al. 2022). Overall, the sensitivity variations of the Stava, Aznalcóllar, and Feijão cases follow a similar trend, except for the yield stress and surface roughness curves, despite the different characteristics of these three failures (Back-analyzed Case Studies section).

The sensitivity to breach width displays decreasing trends in all of the plots. The breach width has a large influence on the model outputs near the breach, but the influence gradually decreases with increasing runout distance (Fig. 8).

The total released volume has a major influence on all the model outputs at different locations along the runout path (> 10% sensitivity values), with an increasing trend that tends to plateau for some of the scenarios (e.g. Fig. 8c, f, g). In the case of Aznolcollar, there is a fluctuation in the sensitivity of modelled maximum flow velocity to the total released volume (Fig. 8b). This may be attributed to the local constriction of the runout path near a highway bridge that crossed the inundation area near the 50% runout location, where the physical constriction controls the velocity more than any other model outputs.

For the Feijão case, the sensitivity to the yield stress displays an increasing trend, with the highest value at 90% of the runout distance (the last measurement location). Model outputs were not sensitive to yield stress for the Stava and Aznalcóllar cases, which may be due to the steep travel path slope along the Stava creek and the low solid concentration of the Aznalcóllar tailings flow, respectively, nor to viscosity for all three cases.

Comparing the three cases, the variation in sensitivity to surface roughness for each model output have different trends. For the Stava case, the sensitivity of the modelled maximum flow depth to surface roughness displays an increasing trend in the first 25% and a decreasing trend for the rest. In contrast, there was a decreasing trend for the Aznalcóllar case and an almost flat trend for the Feijão case (Fig. 8d–f). These differences might be due to distinct topographic conditions, such as steep terrain, sudden elevation changes, or degree of confinement along the path.

In the case of Stava, the results indicate that the modelled maximum flow depth and front flow arrival time were most sensitive to breach width, while exhibiting very low to zero sensitivity to other inputs at the 10% of runout distance. This sensitivity pattern is similar to what was explained in Sensitivity Analysis Section for the Cadia case, suggesting that it may be due to the proximity of the measurement (10% of runout) to the breach location where the dynamic effects such as rapid changes in material behaviour can be significant. When comparing the three cases, the first 10% of the runout distance for the Stava is less than 500 m from the breach while this distance is about 3 km for Aznalcóllar and 1 km for Feijão. This sensitivity pattern changes at further locations along the runout path (Fig. 8d–g).

In this study, the 50% runout distance of Zone 1 was selected as a reference point to compare general model sensitivity estimates. However, analysis of sensitivity variation along the path suggests that the 50% runout distance may not necessarily be a key location of interest in every case. Instead, the location for sensitivity analysis should be chosen based on the specific purpose of the project, particularly considering the locations of elements at risk.

### Demonstration of Probabilistic Prediction Approach

The Merriespruit demonstration case involved modelling the probability distributions of two key parameters: the inundation area and maximum flow velocity at 50% runout distance. These parameters were modelled with respect to each input variable individually and collectively (Fig. 9). In order to roughly compare the predicted results with the observed ones, one of the main sources of uncertainty, the total released volume, was kept as the reported value, as mentioned in the Methodology section. Figure 9b, d show that the output results were over-predicted. The sensitivity analysis results in Fig. 2 showed that yield stress was the top contributor to the sensitivity of inundation area for more than half of the cases. The over-prediction of the results could be due to the lower yield stress selected (63 Pa) compared with the calibrated value (200 Pa) provided in the “Back-analyzed Case Studies” section. Another reason could be the over-estimation of the average breach width value for the simulation, compared to the reported value.

Selection of input parameters, such as total released volume, rheological parameters, and breach geometry, has been a challenge for tailings dam breach-runout forward analysis. Probability density curves can be used by practitioners and modellers to constrain the ranges of estimated model outputs. For example, modellers may use the curves to identify a range of values that are consistent with a certain level of confidence, or to identify the most likely range of values for the output. By doing so, the uncertainties associated with each input variable can be accounted for, and more accurate model predictions can be made.

However, the approximation methodology used to generate the probability density curves has some limitations and assumptions that need to be considered when interpreting the results. One limitation is the use of statistical distributions to model the uncertainty of the input variables. While this can be a useful approximation, it is important to recognize that the choice of distribution may not always accurately reflect the true uncertainty of the input variable. For instance, the assumption of log-normal distribution may not always hold, particularly for extreme events or rare occurrences, which can lead to underestimation or overestimation of the probability of such events. Thus, the probability density curves should be used with caution and in conjunction with other information and expert judgement. Modellers should also be aware of the limitations and assumptions of the approximation methodology, and carefully consider the potential effect of correlated or extreme events that may not be accurately captured by the probability density curves.

### Limitations of the FOSM Method

Although FOSM is a linearization technique, it can be applied to models with non-linear output functions. The FOSM method linearizes the non-linear output function by approximating it as a Taylor series expansion around the mean values of the input variables. Therefore, it assumes that the output can be locally approximated as a linear function near the mean values of the input variables (Lee and Mosalam 2005). However, the FOSM method comes with limitations that should be considered when interpreting the results. It is an approximate method that only considers the first-order and second-moment (i.e. mean and variance), rather than the distribution function, of the input variables. Therefore, it may not work well for highly non-linear systems (Kunstmann et al. 2002). Better precision could be achieved by using higher-order terms from the Taylor series expansion. However, higher orders involve complex mathematics and require additional statistical information, such as skewness and kurtosis, which are not easy to estimate due to insufficient data. Another limitation is that the interaction between input variables is not considered in the FOSM method (Baecher and Christian 2005; Nadim 2007).

In this study, the FOSM approach was applied to three erosional and eight non-erosional case studies. Although the FOSM method is versatile and can be applied to other models, the FOSM results presented in this study are specific to the HEC-RAS numerical model. Our interpretations provide valuable information about HEC-RAS performance for each case study. Although some similar trends were observed, a larger sample size would be needed to draw broader and more robust conclusions, particularly for the erosional breach cases.

## Conclusions

Our study highlights the importance of understanding the uncertainty and sensitivity of model outputs to different input variables for TDB runout modelling, which can help improve the accuracy of risk assessments and mitigation strategies in industry practice. In this study, the FOSM methodology was applied to a database of 11 back-analyzed historical tailings flows to evaluate the uncertainties in TDB runout modelling. Moreover, a sensitivity analysis was conducted to determine the key factors contributing to the sensitivity of the HEC-RAS model outputs, and sensitivity variations were analyzed at different locations along the runout path. We also investigated the potential application of the FOSM method to probabilistic runout modelling in prediction scenarios.

Overall, the uncertainty results and sensitivity estimates showed similar trends in most of the cases. To be able to generate more reliable model results using HEC-RAS: (1) researchers should develop better methods to predict potential release volumes; and (2) practitioners should use expert judgment when estimating potential release volumes and surface roughness values. However, there were some exceptions for each model output and the primary contributors to the sensitivity of the model outputs varied depending on the case study. The Mt. Polley case, for instance, was highly sensitive to breach width for modelled inundation area, maximum flow velocity, and maximum flow depth, potentially due to the site conditions and the use of the Quadratic rheology model, due to the relatively low solid concentration of the Mt. Polley tailings flow. The Cadia Event 2 was also sensitive to breach width for modelled flow velocity, depth, and flow front arrival time. The influence of surface roughness was observed to be less consequential than rheology, potentially due to the high solid concentration of the Cadia tailings and the use of the Quadratic rheology model, instead of other rheological models that were mainly developed for solid materials. These results reinforce that considering site-specific conditions and the selection of appropriate rheological models are crucial for accurate predictions in TDB runout modelling.

We also found that the sensitivity variations along the path for the Stava, Aznalcóllar, and Feijão cases followed similar trends, with decreasing sensitivity to breach width and increasing sensitivity to total released volume for all three cases and increasing sensitivity to yield stress for Feijão. The sensitivity of the model outputs to surface roughness displayed a different trend for each case, which may be due to different topographic conditions along the runout path.

Lastly, the FOSM methodology was proposed as a probabilistic approach to model-based tailings flow runout prediction. A demonstration of the approach was presented to illustrate the potential usefulness of probability density curves in constraining ranges of estimated model outputs in TDBAs.

### Supplementary Information

Below is the link to the electronic supplementary material.

Supplementary file1 (DOCX 118 KB)

## Data Availability

All the model outputs, sensitivity analyses, and uncertainty estimates have been included as supplementary material.
