# Supplementary material for: Analysis of Uncertainty and Sensitivity in Tailings Dam Breach-Runout Numerical Modelling
Source: Mine Water Environ. 2024 Feb 21;43(1):87–103. doi: 10.1007/s10230-024-00970-w (PMC11045442; doi:10.1007/s10230-024-00970-w)
Supplement: Supplementary file 1 — Supplementary file1 (DOCX 118 KB) [file 10230_2024_970_MOESM1_ESM.docx]

Analysis of uncertainty and sensitivity in tailings dam breach-runout numerical modelling

Negar Ghahramani^1,2^, Daniel A. M. Adria^1,3^, Nahyan M. Rana^4^, Marcelo Llano-Serna^5^, Scott McDougall^1^, Stephen G. Evans^6^ and W. Andy Take^7^

*^1^Department of Earth, Ocean and Atmospheric Sciences, The University of British Columbia, Vancouver, Canada.*

*^2^WSP, Lakewood, CO, USA.*

*^3^Knight Piésold, Vancouver, BC, Canada^.^*

*^4^Klohn Crippen Berger, Toronto, ON, Canada.*

*^5^Red Earth Engineering, Brisbane, Australia.*

*^6^Department of Earth and Environmental Sciences, University of Waterloo, ON, Canada.*

*^7^Department of Civil Engineering, Queen’s University, Kingston, ON, Canada.*

*Corresponding Author: Negar Ghahramani Email: nghahramani@eoas.ubc.ca*

- Additional numerical modelling results

Table A‑1. List of modeled outputs for the Stava TDB, Italy, including the best-fit (BF) and additional 10 runs. TRV represents the reported total released volume, YS represents the best-fit yield stress, V represents the best-fit viscosity, R represents the estimated surface roughness, and TBW represents the reported top breach width. See Table 4-5 for the corresponding values.

| Run name | Run Description | Inundation Area (m^2^) | 50% of Modelled Runout Distance | | |
| --- | --- | --- | --- | --- | --- |
|  |  |  | Max Velocity (m/s) | Max Depth (m) | Arrival Time (s) |
| Run 10 | –10% of BF TBW | 513,429 | 17.92 | 5.95 | 208 |
| Run 8 | –10% of BF R | 544,012 | 19.19 | 6.81 | 193 |
| Run 6 | –10% of BF V | 522,482 | 18.23 | 6.20 | 202 |
| Run 4 | –10% of BF YS | 522,618 | 18.24 | 6.21 | 202 |
| Run 2 | –10% of BF TRV | 505,976 | 17.69 | 5.82 | 205 |
| Best-fit (BF) | - | 522,608 | 18.23 | 6.25 | 202 |
| Run 1 | +10% of BF TRV | 539,171 | 18.61 | 6.52 | 200 |
| Run 3 | +10% of BF YS | 522,575 | 18.23 | 6.21 | 202 |
| Run 5 | +10% of BF V | 522,353 | 18.23 | 6.22 | 202 |
| Run 7 | +10% of BF R | 504,003 | 17.16 | 5.59 | 210 |
| Run 9 | +10% of BF TBW | 536,790 | 18.32 | 6.31 | 198 |

Table A‑2. List of modeled outputs for the Stava TDB, Italy, including the best-fit and additional 10 runs at various locations along the flow path

| Run name | 10% of Modelled Runout Distance | | | 25% of Modelled Runout Distance | | | 75% of Modelled Runout Distance | | | 90% of Modelled Runout Distance | | |
| --- | --- | --- | --- | --- | --- | --- | --- | --- | --- | --- | --- | --- |
|  | Max Velocity (m/s) | Max Depth (m) | Arrival Time (s) | Max Velocity (m/s) | Max Depth (m) | Arrival Time (s) | Max Velocity (m/s) | Max Depth (m) | Arrival Time (s) | Max Velocity (m/s) | Max Depth (m) | Arrival Time (s) |
| Run 10 | 14.48 | 6.54 | 31 | 12.59 | 8.58 | 104 | 8.52 | 4.95 | 325 | 4.94 | 4.71 | 414 |
| Run 8 | 16.52 | 6.84 | 30 | 14.32 | 9.83 | 97 | 9.87 | 5.21 | 304 | 5.34 | 4.72 | 386 |
| Run 6 | 15.28 | 6.95 | 30 | 13.06 | 9.02 | 101 | 8.69 | 5.04 | 318 | 5.00 | 4.74 | 406 |
| Run 4 | 15.28 | 6.95 | 30 | 13.06 | 9.03 | 101 | 8.69 | 5.04 | 318 | 4.99 | 4.74 | 406 |
| Run 2 | 15.20 | 6.95 | 30 | 12.70 | 8.76 | 101 | 8.25 | 4.72 | 325 | 4.79 | 4.55 | 415 |
| Best-fit | 15.28 | 6.95 | 30 | 13.06 | 9.02 | 101 | 8.69 | 5.04 | 318 | 4.99 | 4.74 | 406 |
| Run 1 | 15.34 | 6.96 | 30 | 13.31 | 9.23 | 100 | 9.08 | 5.33 | 313 | 5.18 | 4.86 | 399 |
| Run 3 | 15.28 | 6.95 | 30 | 13.03 | 9.03 | 101 | 8.69 | 5.04 | 318 | 4.99 | 4.74 | 406 |
| Run 5 | 15.28 | 6.97 | 30 | 13.03 | 9.03 | 101 | 8.69 | 5.04 | 318 | 4.99 | 4.74 | 406 |
| Run 7 | 14.16 | 6.90 | 30 | 12.29 | 8.36 | 104 | 7.71 | 4.99 | 334 | 4.68 | 4.68 | 427 |
| Run 9 | 15.85 | 7.69 | 30 | 13.37 | 9.32 | 98 | 8.74 | 5.06 | 314 | 5.01 | 4.72 | 402 |

Table A‑3. List of modeled outputs for the Tapo Canyon Event 1 TDB, USA, including the best-fit (BF) outputs and additional 10 runs. TRV represents the reported total released volume, YS represents the best-fit yield stress, V represents the best-fit viscosity, R represents the estimated surface roughness, and TBW represents the reported top breach width. See Table 4-5 for the corresponding values.

| Run name | Run Description | Inundation Area (m^2^) | 50% of Modelled Runout Distance | | |
| --- | --- | --- | --- | --- | --- |
|  |  |  | Max Velocity (m/s) | Max Depth (m) | Arrival Time (s) |
| Run 10 | –10% of BF TBW | 18,799 | 6.30 | 3.66 | 19.0 |
| Run 8 | –10% of BF R | 19,473 | 6.88 | 4.02 | 18.6 |
| Run 6 | –10% of BF V | 19,139 | 6.57 | 3.85 | 18.8 |
| Run 4 | –10% of BF YS | 20,210 | 6.87 | 3.96 | 18.6 |
| Run 2 | –10% of BF TRV | 18,035 | 6.29 | 3.66 | 19.0 |
| Best-fit (BF) | - | 19,136 | 6.57 | 3.84 | 18.8 |
| Run 1 | +10% of BF TRV | 20,167 | 6.81 | 4.01 | 18.4 |
| Run 3 | +10% of BF YS | 18,149 | 6.26 | 3.72 | 19.0 |
| Run 5 | +10% of BF V | 19,133 | 6.56 | 3.84 | 18.8 |
| Run 7 | +10% of BF R | 18,871 | 6.27 | 3.68 | 19.0 |
| Run 9 | +10% of BF TBW | 19,349 | 6.66 | 3.90 | 18.4 |

Table A‑4. List of modeled outputs for the Merriespruit TDB, South Africa, including the best-fit (BF) outputs and additional 10 runs. TRV represents the reported total released volume, YS represents the best-fit yield stress, V represents the best-fit viscosity, R represents the estimated surface roughness, and TBW represents the reported top breach width. See Table 4-5 for the corresponding values.

| Run name | Run Description | Inundation Area (m^2^) | 50% of Modelled Runout Distance | | |
| --- | --- | --- | --- | --- | --- |
|  |  |  | Max Velocity (m/s) | Max Depth (m) | Arrival Time (s) |
| Run 10 | –10% of BF TBW | 1,003,647 | 2.04 | 1.92 | 256 |
| Run 8 | –10% of BF R | 1,017,722 | 2.30 | 1.96 | 230 |
| Run 6 | –10% of BF V | 1,021,908 | 2.09 | 1.97 | 249 |
| Run 4 | –10% of BF YS | 1,056,117 | 2.14 | 1.97 | 245 |
| Run 2 | –10% of BF TRV | 967,889 | 2.00 | 1.88 | 256 |
| Best-fit (BF) | - | 1,021,109 | 2.09 | 1.97 | 249 |
| Run 1 | +10% of BF TRV | 1,070,006 | 2.17 | 2.05 | 243 |
| Run 3 | +10% of BF YS | 989,445 | 2.04 | 1.96 | 252 |
| Run 5 | +10% of BF V | 1,019,565 | 2.09 | 1.97 | 249 |
| Run 7 | +10% of BF R | 1,025,489 | 1.92 | 1.98 | 268 |
| Run 9 | +10% of BF TBW | 1,041,511 | 2.12 | 1.99 | 244 |

Table A‑5. List of modeled outputs for the Aznalcollar TDB, Spain, including the best-fit (BF) outputs and additional 12 runs. TRV represents the reported total released volume, YS represents the best-fit yield stress, V represents the best-fit viscosity, R represents the estimated surface roughness, TBW represents the reported top breach width, and BFT represents the estimated breach formation time. See Table 4-5 for the corresponding values.

| Run name | Run Description | Inundation Area (m^2^) | 50% of Modelled Runout Distance | | |
| --- | --- | --- | --- | --- | --- |
|  |  |  | Max Velocity (m/s) | Max Depth (m) | Arrival Time (s) |
| Run 12 | –10% of BF BFT | 15,485,859 | 1.26 | 3.83 | 12234 |
| Run 10 | –10% of BF TBW | 15,290,659 | 1.25 | 3.83 | 12846 |
| Run 8 | –10% of BF R | 15,307,551 | 1.37 | 3.82 | 11676 |
| Run 6 | –10% of BF V | 15,430,365 | 1.26 | 3.83 | 12462 |
| Run 4 | –10% of BF YS | 15,411,678 | 1.26 | 3.83 | 12468 |
| Run 2 | –10% of BF TRV | 14,224,502 | 1.25 | 3.71 | 12714 |
| Best-fit (BF) | - | 15,419,072 | 1.26 | 3.83 | 12468 |
| Run 1 | +10% of BF TRV | 16,423,275 | 1.27 | 3.94 | 12252 |
| Run 3 | +10% of BF YS | 15,418,709 | 1.26 | 3.83 | 12468 |
| Run 5 | +10% of BF V | 15,406,966 | 1.26 | 3.83 | 12474 |
| Run 7 | +10% of BF R | 15,516,288 | 1.16 | 3.84 | 13260 |
| Run 9 | +10% of BF TBW | 15,515,424 | 1.26 | 3.84 | 12174 |
| Run 11 | +10% of BF BFT | 15,352,970 | 1.26 | 3.83 | 12690 |

Table A‑6. List of modeled outputs for the Aznalcollar TDB, Spain, including the best-fit outputs and additional 12 runs at various locations along the flow path

| Run name | 10% of Modelled Runout Distance | | | 25% of Modelled Runout Distance | | | 75% of Modelled Runout Distance | | | 90% of Modelled Runout Distance | | |
| --- | --- | --- | --- | --- | --- | --- | --- | --- | --- | --- | --- | --- |
|  | Max Velocity (m/s) | Max Depth (m) | Arrival Time (s) | Max Velocity (m/s) | Max Depth (m) | Arrival Time (s) | Max Velocity (m/s) | Max Depth (m) | Arrival Time (s) | Max Velocity (m/s) | Max Depth (m) | Arrival Time (s) |
| Run 12 | 2.01 | 5.17 | 2236 | 1.41 | 4.74 | 5784 | 0.85 | 3.80 | 20670 | 1.10 | 3.49 | 26916 |
| Run 10 | 1.86 | 5.07 | 2352 | 1.37 | 4.70 | 6174 | 0.85 | 3.80 | 21300 | 1.10 | 3.49 | 27558 |
| Run 8 | 2.07 | 5.07 | 2166 | 1.50 | 4.70 | 5616 | 0.93 | 3.80 | 19416 | 1.19 | 3.50 | 25158 |
| Run 6 | 1.95 | 5.13 | 2304 | 1.39 | 4.73 | 5928 | 0.85 | 3.80 | 20892 | 1.10 | 3.49 | 27126 |
| Run 4 | 1.95 | 5.13 | 2304 | 1.39 | 4.73 | 5928 | 0.85 | 3.80 | 20922 | 1.10 | 3.49 | 27168 |
| Run 2 | 1.93 | 5.00 | 2322 | 1.36 | 4.58 | 5982 | 0.83 | 3.68 | 21252 | 1.07 | 3.30 | 27576 |
| Best-fit | 1.95 | 5.13 | 2304 | 1.39 | 4.73 | 5928 | 0.85 | 3.80 | 20916 | 1.10 | 3.49 | 27156 |
| Run 1 | 1.97 | 5.25 | 2280 | 1.43 | 4.85 | 5880 | 0.88 | 3.92 | 20616 | 1.12 | 3.66 | 26766 |
| Run 3 | 1.95 | 5.13 | 2304 | 1.39 | 4.73 | 5928 | 0.85 | 3.80 | 20916 | 1.10 | 3.49 | 27156 |
| Run 5 | 1.95 | 5.13 | 2304 | 1.39 | 4.73 | 5928 | 0.85 | 3.80 | 20922 | 1.10 | 3.49 | 27168 |
| Run 7 | 1.84 | 5.18 | 2436 | 1.30 | 4.74 | 6252 | 0.79 | 3.80 | 22410 | 1.02 | 3.48 | 29178 |
| Run 9 | 2.03 | 5.17 | 2250 | 1.41 | 4.74 | 5718 | 0.85 | 3.80 | 20604 | 1.10 | 3.49 | 26844 |
| Run 11 | 1.89 | 5.10 | 2340 | 1.38 | 4.71 | 6096 | 0.85 | 3.80 | 21138 | 1.08 | 3.49 | 27378 |

Table A‑7. List of modeled outputs for the Ajka TDB, Hungary, including the best-fit (BF) outputs and additional 12 runs. TRV represents the reported total released volume, YS represents the best-fit yield stress, V represents the best-fit viscosity, R represents the estimated surface roughness, TBW represents the reported top breach width, and BFT represents the estimated breach formation time. See Table 4-5 for the corresponding values.

| Run name | Run Description | Inundation Area (m^2^) | 50% of Modelled Runout Distance | | |
| --- | --- | --- | --- | --- | --- |
|  |  |  | Max Velocity (m/s) | Max Depth (m) | Arrival Time (s) |
| Run 12 | –10% of BF BFT | 7,061,596 | 0.82 | 0.58 | 20,945 |
| Run 10 | –10% of BF TBW | 7,036,203 | 0.82 | 0.58 | 21,090 |
| Run 8 | –10% of BF R | 7,036,833 | 0.87 | 0.58 | 19,880 |
| Run 6 | –10% of BF V | 7,101,570 | 0.85 | 0.59 | 20,340 |
| Run 4 | –10% of BF YS | 7,137,457 | 0.84 | 0.59 | 20,675 |
| Run 2 | –10% of BF TRV | 6,617,757 | 0.73 | 0.52 | 23,380 |
| Best-fit (BF) | - | 7,050,620 | 0.82 | 0.58 | 21,010 |
| Run 1 | +10% of BF TRV | 7,539,502 | 0.90 | 0.64 | 19,275 |
| Run 3 | +10% of BF YS | 6,974,357 | 0.80 | 0.58 | 21,360 |
| Run 5 | +10% of BF V | 6,999,957 | 0.79 | 0.58 | 21,695 |
| Run 7 | +10% of BF R | 7,057,403 | 0.78 | 0.59 | 22,170 |
| Run 9 | +10% of BF TBW | 7,045,671 | 0.82 | 0.58 | 21,010 |
| Run 11 | +10% of BF BFT | 7,046,073 | 0.82 | 0.58 | 21,045 |

Table A‑8. List of modeled outputs for the Kayakari TDB, Japan, including the best-fit (BF) outputs and additional 10 runs. TRV represents the reported total released volume, YS represents the best-fit yield stress, V represents the best-fit viscosity, R represents the estimated surface roughness, and TBW represents the reported top breach width. See Table 4-5 for the corresponding values.

| Run name | Run Description | Inundation Area (m^2^) | 50% of Modelled Runout Distance | | |
| --- | --- | --- | --- | --- | --- |
|  |  |  | Max Velocity (m/s) | Max Depth (m) | Arrival Time (s) |
| Run 10 | –10% of BF TBW | 107,480 | 5.90 | 1.90 | 150 |
| Run 8 | –10% of BF R | 108,482 | 6.30 | 2.02 | 142 |
| Run 6 | –10% of BF V | 108,228 | 6.02 | 1.94 | 147 |
| Run 4 | –10% of BF YS | 109,493 | 6.07 | 1.94 | 146 |
| Run 2 | –10% of BF TRV | 103,710 | 5.70 | 1.83 | 151 |
| Best-fit (BF) | - | 108,094 | 5.98 | 1.93 | 147 |
| Run 1 | +10% of BF TRV | 111,301 | 6.23 | 2.02 | 145 |
| Run 3 | +10% of BF YS | 106,553 | 5.88 | 1.92 | 148 |
| Run 5 | +10% of BF V | 108,001 | 5.93 | 1.92 | 148 |
| Run 7 | +10% of BF R | 107,953 | 5.68 | 1.88 | 153 |
| Run 9 | +10% of BF TBW | 108,676 | 6.04 | 1.96 | 145 |

Table A‑9. List of modeled outputs for the Mount Polley TDB, Canada, including the best-fit (BF) outputs and additional 12 runs. TRV represents the reported total released volume, YS represents the best-fit yield stress, V represents the best-fit viscosity, R represents the estimated surface roughness, TBW represents the reported top breach width, and BFT represents the estimated breach formation time. See Table 4-5 for the corresponding values.

| Run name | Run Description | Inundation Area (m^2^) | 50% of Modelled Runout Distance | | |
| --- | --- | --- | --- | --- | --- |
|  |  |  | Max Velocity (m/s) | Max Depth (m) | Arrival Time (s) |
| Run 12 | –10% of BF BFT | 3,075,688 | 6.41 | 11.51 | 1980 |
| Run 10 | –10% of BF TBW | 2,750,196 | 5.46 | 10.71 | 1980 |
| Run 8 | –10% of BF R | 2,929,554 | 6.32 | 11.21 | 1880 |
| Run 6 | –10% of BF V | 2,987,185 | 6.16 | 11.29 | 1980 |
| Run 4 | –10% of BF YS | 2,987,280 | 6.16 | 11.29 | 1980 |
| Run 2 | –10% of BF TRV | 2,897,786 | 5.90 | 11.08 | 2000 |
| Best-fit (BF) | - | 2,987,242 | 6.16 | 11.29 | 1980 |
| Run 1 | +10% of BF TRV | 3,070,238 | 6.39 | 11.49 | 1980 |
| Run 3 | +10% of BF YS | 2,987,341 | 6.16 | 11.29 | 1980 |
| Run 5 | +10% of BF V | 2,987,306 | 6.16 | 11.29 | 1980 |
| Run 7 | +10% of BF R | 3,045,088 | 6.00 | 11.38 | 2100 |
| Run 9 | +10% of BF TBW | 3,411,993 | 7.45 | 12.46 | 1980 |
| Run 11 | +10% of BF BFT | 2,904,682 | 5.91 | 11.09 | 1980 |

Table A‑10. List of modeled outputs for the Fundão TDB, Brazil, including the best-fit (BF) outputs and additional 10 runs. TRV represents the reported total released volume, YS represents the best-fit yield stress, V represents the best-fit viscosity, R represents the estimated surface roughness, and TBW represents the reported top breach width. See Table 4-5 for the corresponding values.

| Run name | Run Description | Inundation Area (m^2^) | 50% of Modelled Runout Distance | | |
| --- | --- | --- | --- | --- | --- |
|  |  |  | Max Velocity (m/s) | Max Depth (m) | Arrival Time (s) |
| Run 10 | –10% of BF TBW | 10,126,735 | 2.48 | 14.13 | 6,490 |
| Run 8 | –10% of BF R | 10,210,653 | 2.60 | 14.14 | 6,180 |
| Run 6 | –10% of BF V | 10,216,894 | 2.49 | 14.16 | 6,460 |
| Run 4 | –10% of BF YS | 10,217,599 | 2.49 | 14.17 | 6,460 |
| Run 2 | –10% of BF TRV | 9,803,739 | 2.39 | 13.30 | 6,700 |
| Best-fit (BF) | - | 10,216,937 | 2.49 | 14.16 | 6,460 |
| Run 1 | +10% of BF TRV | 10,605,511 | 2.58 | 14.95 | 6,250 |
| Run 3 | +10% of BF YS | 10,215,233 | 2.49 | 14.16 | 6,470 |
| Run 5 | +10% of BF V | 10,216,907 | 2.49 | 14.16 | 6,460 |
| Run 7 | +10% of BF R | 10,224,774 | 2.38 | 14.19 | 6,760 |
| Run 9 | +10% of BF TBW | 10,216,760 | 2.48 | 14.11 | 6,500 |

Table A‑11. List of modeled outputs for the Tonglvshan TDB, China, including the best-fit (BF) outputs and additional 10 runs. TRV represents the reported total released volume, YS represents the best-fit yield stress, V represents the best-fit viscosity, R represents the estimated surface roughness, and TBW represents the reported top breach width. See Table 4-5 for the corresponding values.

| Run name | Run Description | Inundation Area (m^2^) | 50% of Modelled Runout Distance | | |
| --- | --- | --- | --- | --- | --- |
|  |  |  | Max Velocity (m/s) | Max Depth (m) | Arrival Time (s) |
| Run 10 | –10% of BF TBW | 277,581 | 5.87 | 3.16 | 36.6 |
| Run 8 | –10% of BF R | 279,307 | 6.81 | 3.26 | 33.2 |
| Run 6 | –10% of BF V | 278,801 | 6.27 | 3.18 | 34.4 |
| Run 4 | –10% of BF YS | 283,656 | 6.33 | 3.16 | 34.4 |
| Run 2 | –10% of BF TRV | 263,920 | 6.16 | 2.86 | 34.6 |
| Best-fit (BF) | - | 278,761 | 6.27 | 3.18 | 34.6 |
| Run 1 | +10% of BF TRV | 296,464 | 6.36 | 3.43 | 34.4 |
| Run 3 | +10% of BF YS | 274,323 | 6.21 | 3.20 | 34.6 |
| Run 5 | +10% of BF V | 278,724 | 6.26 | 3.18 | 34.6 |
| Run 7 | +10% of BF R | 279,010 | 5.80 | 3.11 | 36.0 |
| Run 9 | +10% of BF TBW | 279,872 | 6.80 | 3.13 | 33.4 |

Table A‑12. List of modeled outputs for the Cadia Event 2 TDB, Australia, including the best-fit (BF) outputs and additional 10 runs. TRV represents the reported total released volume, YS represents the best-fit yield stress, V represents the best-fit viscosity, R represents the estimated surface roughness, and TBW represents the reported top breach width. See Table 4-5 for the corresponding values.

| Run name | Run Description | Inundation Area (m^2^) | 50% of Modelled Runout Distance | | |
| --- | --- | --- | --- | --- | --- |
|  |  |  | Max Velocity (m/s) | Max Depth (m) | Arrival Time (s) |
| Run 10 | –10% of BF TBW | 101,549 | 4.65 | 4.05 | 38 |
| Run 8 | –10% of BF R | 103,188 | 5.26 | 4.23 | 36 |
| Run 6 | –10% of BF V | 103,441 | 5.54 | 4.24 | 35 |
| Run 4 | –10% of BF YS | 106,950 | 5.36 | 4.22 | 35 |
| Run 2 | –10% of BF TRV | 96,461 | 5.01 | 4.08 | 36 |
| Best-fit (BF) | - | 103,077 | 5.20 | 4.23 | 36 |
| Run 1 | +10% of BF TRV | 109,216 | 6.34 | 4.34 | 35 |
| Run 3 | +10% of BF YS | 99,388 | 5.03 | 4.22 | 36 |
| Run 5 | +10% of BF V | 102,670 | 4.87 | 4.18 | 37 |
| Run 7 | +10% of BF R | 103,089 | 5.13 | 4.20 | 36 |
| Run 9 | +10% of BF TBW | 104,086 | 5.69 | 4.35 | 34 |

Table A‑13. List of modeled outputs for the Feijão TDB, Brazil, including the best-fit (BF) outputs and additional 10 runs. TRV represents the reported total released volume, YS represents the best-fit yield stress, V represents the best-fit viscosity, R represents the estimated surface roughness, and TBW represents the reported top breach width. See Table 4-5 for the corresponding values.

| Run name | Run Description | Inundation Area (m^2^) | 50% of Modelled Runout Distance | | |
| --- | --- | --- | --- | --- | --- |
|  |  |  | Max Velocity (m/s) | Max Depth (m) | Arrival Time (s) |
| Run 10 | –10% of BF TBW | 3,606,591 | 5.93 | 9.38 | 699 |
| Run 8 | –10% of BF R | 3,721,218 | 6.46 | 9.36 | 639 |
| Run 6 | –10% of BF V | 3,701,937 | 5.99 | 9.41 | 678 |
| Run 4 | –10% of BF YS | 3,728,632 | 6.05 | 9.38 | 675 |
| Run 2 | –10% of BF TRV | 3,531,426 | 5.63 | 8.88 | 705 |
| Best-fit (BF) | - | 3,700,751 | 5.98 | 9.41 | 679 |
| Run 1 | +10% of BF TRV | 3,853,229 | 6.31 | 9.92 | 657 |
| Run 3 | +10% of BF YS | 3,671,358 | 5.93 | 9.43 | 683 |
| Run 5 | +10% of BF V | 3,700,025 | 5.98 | 9.41 | 679 |
| Run 7 | +10% of BF R | 3,680,417 | 5.47 | 9.47 | 719 |
| Run 9 | +10% of BF TBW | 3,756,398 | 5.99 | 9.41 | 670 |

Table A‑14. List of modeled outputs for the Feijão TDB, Brazil and additional 10 runs at various locations along the flow path

| Run name | 10% of Modelled Runout Distance | | | 25% of Modelled Runout Distance | | | 75% of Modelled Runout Distance | | | 90% of Modelled Runout Distance | | |
| --- | --- | --- | --- | --- | --- | --- | --- | --- | --- | --- | --- | --- |
|  | Max Velocity (m/s) | Max Depth (m) | Arrival Time (s) | Max Velocity (m/s) | Max Depth (m) | Arrival Time (s) | Max Velocity (m/s) | Max Depth (m) | Arrival Time (s) | Max Velocity (m/s) | Max Depth (m) | Arrival Time (s) |
| Run 10 | 14.41 | 17.01 | 78 | 7.38 | 17.38 | 205 | 3.14 | 10.71 | 1527 | 3.42 | 10.85 | 2075 |
| Run 8 | 16.12 | 18.38 | 75 | 8.73 | 17.55 | 189 | 3.43 | 10.69 | 1406 | 3.71 | 10.89 | 1916 |
| Run 6 | 15.15 | 18.28 | 76 | 7.83 | 17.64 | 196 | 3.15 | 10.73 | 1490 | 3.44 | 10.86 | 2040 |
| Run 4 | 15.16 | 18.28 | 76 | 7.85 | 17.67 | 198 | 3.21 | 10.83 | 1478 | 3.50 | 10.94 | 2011 |
| Run 2 | 14.78 | 17.55 | 76 | 7.46 | 16.75 | 200 | 2.93 | 9.98 | 1585 | 3.26 | 10.25 | 2175 |
| Best-fit | 15.13 | 18.26 | 76 | 7.82 | 17.63 | 196 | 3.14 | 10.72 | 1493 | 3.43 | 10.85 | 2044 |
| Run 1 | 15.46 | 18.88 | 75 | 8.16 | 18.44 | 193 | 3.34 | 11.42 | 1422 | 3.59 | 11.43 | 1938 |
| Run 3 | 15.13 | 18.26 | 76 | 7.80 | 17.59 | 197 | 3.08 | 10.64 | 1517 | 3.36 | 10.77 | 2078 |
| Run 5 | 15.15 | 18.27 | 75 | 7.83 | 17.63 | 196 | 3.14 | 10.72 | 1499 | 3.43 | 10.86 | 2046 |
| Run 7 | 14.18 | 18.41 | 77 | 7.11 | 17.70 | 204 | 2.91 | 10.74 | 1591 | 3.19 | 10.83 | 2175 |
| Run 9 | 15.52 | 18.86 | 73 | 8.08 | 17.69 | 191 | 3.14 | 10.71 | 1487 | 3.43 | 10.84 | 2033 |

- Uncertainty and Sensitivity Results

Table B‑1 Estimated sensitivity, uncertainty and weight in global variance of modelled inundation area with respect to the listed outputs for the Stava TDB, Italy.

| **Input** | **Sensitivity Measurement (%)** | **Uncertainty (CoV) (%)** | **Weight in Global Variance (%)** |
| --- | --- | --- | --- |
| Total released volume (M m3) | 34.31% | 22.23% | 24.25% |
| Yield Strength (Pa) | 0.04% | 0.07% | 0.00% |
| Viscosity (Pa.s) | 0.13% | 0.15% | 0.00% |
| Roughness | 41.36% | 28.71% | 40.45% |
| Top Breach Width (m) | 24.15% | 26.82% | 35.30% |

Table B‑2 Estimated sensitivity, uncertainty and weight in global variance of modelled maximum flow velocity at 50% runout with respect to the listed outputs for the Stava TDB, Italy.

| **Input** | **Sensitivity Measurement (%)** | **Uncertainty (CoV) (%)** | **Weight in Global Variance (%)** |
| --- | --- | --- | --- |
| Total released volume (M m3) | 27.38% | 17.66% | 14.00% |
| Yield Strength (Pa) | 0.30% | 0.44% | 0.01% |
| Viscosity (Pa.s) | 0.00% | 0.00% | 0.00% |
| Roughness | 60.42% | 41.76% | 78.22% |
| Top Breach Width (m) | 11.90% | 13.17% | 7.77% |

Table B‑3 Estimated sensitivity, uncertainty and weight in global variance of modelled maximum flow depth at 50% runout with respect to the listed outputs for the Stava TDB, Italy.

| **Input** | **Sensitivity Measurement (%)** | **Uncertainty (CoV) (%)** | **Weight in Global Variance (%)** |
| --- | --- | --- | --- |
| Total released volume (M m3) | 30.43% | 39.20% | 18.99% |
| Yield Strength (Pa) | 0.00% | 0.00% | 0.00% |
| Viscosity (Pa.s) | 0.87% | 1.92% | 0.05% |
| Roughness | 53.04% | 73.20% | 66.21% |
| Top Breach Width (m) | 15.65% | 34.56% | 14.76% |

Table B‑4 Estimated sensitivity, uncertainty and weight in global variance of modelled flow front arrival time at 50% runout with respect to the listed outputs for the Stava TDB, Italy.

| **Input** | **Sensitivity Measurement (%)** | **Uncertainty (CoV) (%)** | **Weight in Global Variance (%)** |
| --- | --- | --- | --- |
| Total released volume (M m3) | 15.63% | 8.66% | 3.84% |
| Yield Strength (Pa) | 0.00% | 0.00% | 0.00% |
| Viscosity (Pa.s) | 0.00% | 0.00% | 0.00% |
| Roughness | 53.13% | 31.56% | 50.99% |
| Top Breach Width (m) | 31.25% | 29.70% | 45.17% |

Table B‑5 Estimated sensitivity, uncertainty and weight in global variance of modelled inundation area with respect to the listed outputs for the Tapo Canyon, USA, Event 1 TDB.

| **Input** | **Sensitivity Measurement (%)** | **Uncertainty (CoV) (%)** | **Weight in Global Variance (%)** |
| --- | --- | --- | --- |
| Total released volume (M m3) | 39.84% | 38.99% | 17.93% |
| Yield Strength (Pa) | 38.52% | 80.78% | 76.93% |
| Viscosity (Pa.s) | 0.11% | 0.06% | 0.00% |
| Roughness | 11.25% | 11.80% | 1.64% |
| Top Breach Width (m) | 10.28% | 17.24% | 3.51% |

Table B‑6 Estimated sensitivity, uncertainty and weight in global variance of modelled maximum flow velocity at 50% runout with respect to the listed outputs for the Tapo Canyon, USA, Event 1 TDB.

| **Input** | **Sensitivity Measurement (%)** | **Uncertainty (CoV) (%)** | **Weight in Global Variance (%)** |
| --- | --- | --- | --- |
| Total released volume (M m3) | 24.64% | 27.70% | 9.70% |
| Yield Strength (Pa) | 28.91% | 69.63% | 61.31% |
| Viscosity (Pa.s) | 0.47% | 0.30% | 0.00% |
| Roughness | 28.91% | 34.82% | 15.33% |
| Top Breach Width (m) | 17.06% | 32.88% | 13.67% |

Table B‑7 Estimated sensitivity, uncertainty and weight in global variance of modelled maximum flow depth at 50% runout with respect to the listed outputs for the Tapo Canyon, USA, Event 1 TDB.

| **Input** | **Sensitivity Measurement (%)** | **Uncertainty (CoV) (%)** | **Weight in Global Variance (%)** |
| --- | --- | --- | --- |
| Total released volume (M m3) | 30.43% | 39.20% | 18.99% |
| Yield Strength (Pa) | 29.66% | 31.90% | 17.78% |
| Viscosity (Pa.s) | 20.34% | 46.88% | 38.39% |
| Roughness | 0.85% | 0.52% | 0.00% |
| Top Breach Width (m) | 28.81% | 33.20% | 19.26% |

Table B‑8 Estimated sensitivity, uncertainty and weight in global variance of modelled flow front arrival time at 50% runout with respect to the listed outputs for the Tapo Canyon, USA, Event 1 TDB.

| **Input** | **Sensitivity Measurement (%)** | **Uncertainty (CoV) (%)** | **Weight in Global Variance (%)** |
| --- | --- | --- | --- |
| Total released volume (M m3) | 30.00% | 11.17% | 15.41% |
| Yield Strength (Pa) | 20.00% | 15.96% | 31.45% |
| Viscosity (Pa.s) | 0.00% | 0.00% | 0.00% |
| Roughness | 20.00% | 7.98% | 7.86% |
| Top Breach Width (m) | 30.00% | 19.15% | 45.28% |

Table B‑9 Estimated sensitivity, uncertainty and weight in global variance of modelled inundation area with respect to the listed inputs for the Merriespruit TDB, South Africa.

| **Input** | **Sensitivity Measurement (%)** | **Uncertainty (CoV) (%)** | **Weight in Global Variance (%)** |
| --- | --- | --- | --- |
| Total released volume (M m3) | 47% | 35% | 30% |
| Yield Strength (Pa) | 31% | 49% | 58% |
| Viscosity (Pa.s) | 1% | 0% | 0% |
| Roughness | 4% | 3% | 0% |
| Top Breach Width (m) | 17% | 22% | 12% |

Table B‑10 Estimated sensitivity, uncertainty and weight in global variance of modelled maximum flow velocity at 50% runout with respect to the listed inputs for the Merriespruit TDB, South Africa.

| **Input** | **Sensitivity Measurement (%)** | **Uncertainty (CoV) (%)** | **Weight in Global Variance (%)** |
| --- | --- | --- | --- |
| Total released volume (M m3) | 23% | 28% | 11% |
| Yield Strength (Pa) | 14% | 36% | 18% |
| Viscosity (Pa.s) | 0% | 0% | 0% |
| Roughness | 52% | 68% | 64% |
| Top Breach Width (m) | 11% | 23% | 7% |

Table B‑11 Estimated sensitivity, uncertainty and weight in global variance of modelled maximum flow depth at 50% runout with respect to the listed inputs for the Merriespruit TDB, South Africa.

| **Input** | **Sensitivity Measurement (%)** | **Uncertainty (CoV) (%)** | **Weight in Global Variance (%)** |
| --- | --- | --- | --- |
| Total released volume (M m3) | 63% | 30% | 65% |
| Yield Strength (Pa) | 4% | 4% | 1% |
| Viscosity (Pa.s) | 0% | 0% | 0% |
| Roughness | 7% | 4% | 1% |
| Top Breach Width (m) | 26% | 21% | 33% |

Table B‑12 Estimated sensitivity, uncertainty and weight in global variance of modelled flow front arrival time at 50% runout with respect to the listed inputs for the Merriespruit TDB, South Africa.

| **Input** | **Sensitivity Measurement (%)** | **Uncertainty (CoV) (%)** | **Weight in Global Variance (%)** |
| --- | --- | --- | --- |
| Total released volume (M m3) | 19% | 18% | 7% |
| Yield Strength (Pa) | 10% | 21% | 9% |
| Viscosity (Pa.s) | 0% | 0% | 0% |
| Roughness | 54% | 57% | 67% |
| Top Breach Width (m) | 17% | 29% | 17% |

Table B‑13 Estimated sensitivity, uncertainty and weight in global variance of modelled inundation area with respect to the listed inputs for the Aznalcóllar TDB, Spain.

| **Input** | **Sensitivity Measurement (%)** | **Uncertainty (CoV) (%)** | **Weight in Global Variance (%)** |
| --- | --- | --- | --- |
| Total released volume (M m3) | 79% | 50% | 96% |
| Yield Strength (Pa) | 0% | 0% | 0% |
| Viscosity (Pa.s) | 1% | 0% | 0% |
| Roughness | 7% | 5% | 1% |
| Formation Time (hr) | 5% | 4% | 1% |
| Top Breach Width (m) | 8% | 9% | 3% |

Table B‑14 Estimated sensitivity, uncertainty and weight in global variance of modelled maximum flow velocity at 50% runout with respect to the listed inputs for the Aznalcóllar TDB, Spain.

| **Input** | **Sensitivity Measurement (%)** | **Uncertainty (CoV) (%)** | **Weight in Global Variance (%)** |
| --- | --- | --- | --- |
| Total released volume (M m3) | 8% | 6% | 1% |
| Yield Strength (Pa) | 0% | 0% | 0% |
| Viscosity (Pa.s) | 0% | 0% | 0% |
| Roughness | 88% | 63% | 99% |
| Formation Time (hr) | 0% | 0% | 0% |
| Top Breach Width (m) | 4% | 5% | 1% |

Table B‑15 Estimated sensitivity, uncertainty and weight in global variance of modelled maximum flow depth at 50% runout with respect to the listed inputs for the Aznalcóllar TDB, Spain.

| **Input** | **Sensitivity Measurement (%)** | **Uncertainty (CoV) (%)** | **Weight in Global Variance (%)** |
| --- | --- | --- | --- |
| Total released volume (M m3) | 88% | 21% | 99% |
| Yield Strength (Pa) | 0% | 0% | 0% |
| Viscosity (Pa.s) | 0% | 0% | 0% |
| Roughness | 8% | 2% | 1% |
| Formation Time (hr) | 0% | 0% | 0% |
| Top Breach Width (m) | 4% | 2% | 1% |

Table B‑16 Estimated sensitivity, uncertainty and weight in global variance of modelled flow front arrival time at 50% runout with respect to the listed inputs for the Aznalcóllar TDB, Spain.

| **Input** | **Sensitivity Measurement (%)** | **Uncertainty (CoV) (%)** | **Weight in Global Variance (%)** |
| --- | --- | --- | --- |
| Total released volume (M m3) | 15% | 13% | 5% |
| Yield Strength (Pa) | 0% | 0% | 0% |
| Viscosity (Pa.s) | 0% | 0% | 0% |
| Roughness | 50% | 48% | 65% |
| Formation Time (hr) | 14% | 16% | 8% |
| Top Breach Width (m) | 21% | 32% | 30% |

Table B‑17 Estimated sensitivity, uncertainty and weight in global variance of modelled inundation area with respect to the listed inputs for the Ajka TDB, Hungary.

| **Input** | **Sensitivity Measurement (%)** | **Uncertainty (CoV) (%)** | **Weight in Global Variance (%)** |
| --- | --- | --- | --- |
| Total released volume (M m3) | 75% | 46% | 94% |
| Yield Strength (Pa) | 13% | 10% | 5% |
| Viscosity (Pa.s) | 8% | 4% | 1% |
| Roughness | 2% | 1% | 0% |
| Formation Time (hr) | 1% | 1% | 0% |
| Top Breach Width (m) | 1% | 1% | 0% |

Table B‑18 Estimated sensitivity, uncertainty and weight in global variance of modelled maximum flow velocity at 50% runout with respect to the listed inputs for the Ajka TDB, Hungary.

| **Input** | **Sensitivity Measurement (%)** | **Uncertainty (CoV) (%)** | **Weight in Global Variance (%)** |
| --- | --- | --- | --- |
| Total released volume (M m3) | 47% | 73% | 66% |
| Yield Strength (Pa) | 11% | 22% | 6% |
| Viscosity (Pa.s) | 17% | 22% | 6% |
| Roughness | 25% | 41% | 21% |
| Formation Time (hr) | 0% | 0% | 0% |
| Top Breach Width (m) | 0% | 0% | 0% |

Table B‑19 Estimated sensitivity, uncertainty and weight in global variance of modelled maximum flow depth at 50% runout with respect to the listed inputs for the Ajka TDB, Hungary.

| **Input** | **Sensitivity Measurement (%)** | **Uncertainty (CoV) (%)** | **Weight in Global Variance (%)** |
| --- | --- | --- | --- |
| Total released volume (M m3) | 80% | 72% | 98% |
| Yield Strength (Pa) | 7% | 8% | 1% |
| Viscosity (Pa.s) | 7% | 5% | 0% |
| Roughness | 7% | 6% | 1% |
| Formation Time (hr) | 0% | 0% | 0% |
| Top Breach Width (m) | 0% | 0% | 0% |

Table B‑20 Estimated sensitivity, uncertainty and weight in global variance of modelled flow front arrival time at 50% runout with respect to the listed inputs for the Ajka TDB, Hungary.

| **Input** | **Sensitivity Measurement (%)** | **Uncertainty (CoV) (%)** | **Weight in Global Variance (%)** |
| --- | --- | --- | --- |
| Total released volume (M m3) | 48% | 68% | 67% |
| Yield Strength (Pa) | 8% | 15% | 3% |
| Viscosity (Pa.s) | 16% | 19% | 5% |
| Roughness | 27% | 41% | 24% |
| Formation Time (hr) | 1% | 2% | 0% |
| Top Breach Width (m) | 1% | 2% | 0% |

Table B‑21 Estimated sensitivity, uncertainty and weight in global variance of modelled inundation area with respect to the listed inputs for the Kayakari TDB, Japan.

| **Input** | **Sensitivity Measurement (%)** | **Uncertainty (CoV) (%)** | **Weight in Global Variance (%)** |
| --- | --- | --- | --- |
| Total released volume (M m3) | 61% | 25% | 57% |
| Yield Strength (Pa) | 24% | 20% | 39% |
| Viscosity (Pa.s) | 2% | 0% | 0% |
| Roughness | 4% | 2% | 0% |
| Top Breach Width (m) | 10% | 7% | 4% |

Table B‑22 Estimated sensitivity, uncertainty and weight in global variance of modelled maximum flow velocity at 50% runout with respect to the listed inputs for the Kayakari TDB, Japan.

| **Input** | **Sensitivity Measurement (%)** | **Uncertainty (CoV) (%)** | **Weight in Global Variance (%)** |
| --- | --- | --- | --- |
| Total released volume (M m3) | 34% | 31% | 30% |
| Yield Strength (Pa) | 12% | 24% | 17% |
| Viscosity (Pa.s) | 6% | 3% | 0% |
| Roughness | 39% | 39% | 47% |
| Top Breach Width (m) | 9% | 14% | 6% |

Table B‑23 Estimated sensitivity, uncertainty and weight in global variance of modelled maximum flow depth at 50% runout with respect to the listed inputs for the Kayakari TDB, Japan.

| **Input** | **Sensitivity Measurement (%)** | **Uncertainty (CoV) (%)** | **Weight in Global Variance (%)** |
| --- | --- | --- | --- |
| Total released volume (M m3) | 44% | 34% | 51% |
| Yield Strength (Pa) | 5% | 8% | 3% |
| Viscosity (Pa.s) | 5% | 2% | 0% |
| Roughness | 33% | 27% | 32% |
| Top Breach Width (m) | 14% | 19% | 15% |

Table B‑24 Estimated sensitivity, uncertainty and weight in global variance of modelled flow front arrival time at 50% runout with respect to the listed inputs for the Kayakari TDB, Japan.

| **Input** | **Sensitivity Measurement (%)** | **Uncertainty (CoV) (%)** | **Weight in Global Variance (%)** |
| --- | --- | --- | --- |
| Total released volume (M m3) | 24% | 14% | 13% |
| Yield Strength (Pa) | 8% | 10% | 7% |
| Viscosity (Pa.s) | 4% | 1% | 0% |
| Roughness | 44% | 28% | 52% |
| Top Breach Width (m) | 20% | 20% | 28% |

Table B‑25 Estimated sensitivity, uncertainty and weight in global variance of modelled inundation area with respect to the listed inputs for the Mount Polley TDB, Canada.

| **Input** | **Sensitivity Measurement (%)** | **Uncertainty (CoV) (%)** | **Weight in Global Variance (%)** |
| --- | --- | --- | --- |
| Total released volume (M m3) | 15% | 20% | 2% |
| Yield Strength (Pa) | 0% | 0% | 0% |
| Viscosity (Pa.s) | 0% | 0% | 0% |
| Roughness | 10% | 15% | 1% |
| Formation Time (hr) | 15% | 26% | 4% |
| Top Breach Width (m) | 59% | 133% | 96% |

Table B‑26 Estimated sensitivity, uncertainty and weight in global variance of modelled maximum flow velocity at 50% runout with respect to the listed inputs for the Mount Polley TDB, Canada.

| **Input** | **Sensitivity Measurement (%)** | **Uncertainty (CoV) (%)** | **Weight in Global Variance (%)** |
| --- | --- | --- | --- |
| Total released volume (M m3) | 15% | 28% | 2% |
| Yield Strength (Pa) | 0% | 0% | 0% |
| Viscosity (Pa.s) | 0% | 0% | 0% |
| Roughness | 10% | 21% | 1% |
| Formation Time (hr) | 0% | 37% | 3% |
| Top Breach Width (m) | 60% | 194% | 97% |

Table B‑27 Estimated sensitivity, uncertainty and weight in global variance of modelled maximum flow depth at 50% runout with respect to the listed inputs for the Mount PolleyTDB, Canada.

| **Input** | **Sensitivity Measurement (%)** | **Uncertainty (CoV) (%)** | **Weight in Global Variance (%)** |
| --- | --- | --- | --- |
| Total released volume (M m3) | 15% | 13% | 2% |
| Yield Strength (Pa) | 0% | 0% | 0% |
| Viscosity (Pa.s) | 0% | 0% | 0% |
| Roughness | 6% | 6% | 0% |
| Formation Time (hr) | 15% | 17% | 3% |
| Top Breach Width (m) | 64% | 93% | 98% |

Table B‑28 Estimated sensitivity, uncertainty and weight in global variance of modelled flow front arrival time at 50% runout with respect to the listed inputs for the Mount Polley TDB, Canada.

| **Input** | **Sensitivity Measurement (%)** | **Uncertainty (CoV) (%)** | **Weight in Global Variance (%)** |
| --- | --- | --- | --- |
| Total released volume (M m3) | 8% | 4% | 1% |
| Yield Strength (Pa) | 0% | 0% | 0% |
| Viscosity (Pa.s) | 0% | 0% | 0% |
| Roughness | 92% | 44% | 99% |
| Formation Time (hr) | 0% | 0% | 0% |
| Top Breach Width (m) | 0% | 0% | 0% |

Table B‑29 Estimated sensitivity, uncertainty and weight in global variance of modelled inundation area with respect to the listed inputs for the Fundão TDB, Brazil.

| **Input** | **Sensitivity Measurement (%)** | **Uncertainty (CoV) (%)** | **Weight in Global Variance (%)** |
| --- | --- | --- | --- |
| Total released volume (M m3) | 88% | 27% | 96% |
| Yield Strength (Pa) | 0% | 0% | 0% |
| Viscosity (Pa.s) | 0% | 0% | 0% |
| Roughness | 2% | 1% | 0% |
| Top Breach Width (m) | 10% | 5% | 4% |

Table B‑30 Estimated sensitivity, uncertainty and weight in global variance of modelled maximum flow velocity at 50% runout with respect to the listed inputs for the Fundão TDB, Brazil.

| **Input** | **Sensitivity Measurement (%)** | **Uncertainty (CoV) (%)** | **Weight in Global Variance (%)** |
| --- | --- | --- | --- |
| Total released volume (M m3) | 46% | 27% | 39% |
| Yield Strength (Pa) | 0% | 0% | 0% |
| Viscosity (Pa.s) | 0% | 0% | 0% |
| Roughness | 54% | 33% | 61% |
| Top Breach Width (m) | 0% | 0% | 0% |

Table B‑31 Estimated sensitivity, uncertainty and weight in global variance of modelled maximum flow depth at 50% runout with respect to the listed inputs for the Fundão TDB, Brazil.

| **Input** | **Sensitivity Measurement (%)** | **Uncertainty (CoV) (%)** | **Weight in Global Variance (%)** |
| --- | --- | --- | --- |
| Total released volume (M m3) | 95% | 41% | 100% |
| Yield Strength (Pa) | 1% | 1% | 0% |
| Viscosity (Pa.s) | 0% | 0% | 0% |
| Roughness | 3% | 1% | 0% |
| Top Breach Width (m) | 1% | 1% | 0% |

Table B‑32 Estimated sensitivity, uncertainty and weight in global variance of modelled flow front arrival time at 50% runout with respect to the listed inputs for the Fundão TDB, Brazil.

| **Input** | **Sensitivity Measurement (%)** | **Uncertainty (CoV) (%)** | **Weight in Global Variance (%)** |
| --- | --- | --- | --- |
| Total released volume (M m3) | 43% | 24% | 34% |
| Yield Strength (Pa) | 1% | 1% | 0% |
| Viscosity (Pa.s) | 0% | 0% | 0% |
| Roughness | 55% | 34% | 66% |
| Top Breach Width (m) | 1% | 1% | 0% |

Table B‑33 Estimated sensitivity, uncertainty and weight in global variance of modelled inundation area with respect to the listed inputs for the Tonglvshan TDB, China.

| **Input** | **Sensitivity Measurement (%)** | **Uncertainty (CoV) (%)** | **Weight in Global Variance (%)** |
| --- | --- | --- | --- |
| Total released volume (M m3) | 73% | 41% | 72% |
| Yield Strength (Pa) | 21% | 25% | 27% |
| Viscosity (Pa.s) | 0% | 0% | 0% |
| Roughness | 1% | 0% | 0% |
| Top Breach Width (m) | 5% | 5% | 1% |

Table B‑34 Estimated sensitivity, uncertainty and weight in global variance of modelled maximum flow velocity at 50% runout with respect to the listed inputs for the Tonglvshan TDB, China.

| **Input** | **Sensitivity Measurement (%)** | **Uncertainty (CoV) (%)** | **Weight in Global Variance (%)** |
| --- | --- | --- | --- |
| Total released volume (M m3) | 9% | 11% | 1% |
| Yield Strength (Pa) | 5% | 14% | 2% |
| Viscosity (Pa.s) | 0% | 0% | 0% |
| Roughness | 44% | 60% | 31% |
| Top Breach Width (m) | 41% | 89% | 67% |

Table B‑35 Estimated sensitivity, uncertainty and weight in global variance of modelled maximum flow depth at 50% runout with respect to the listed inputs for the Tonglvshan TDB, China.

| **Input** | **Sensitivity Measurement (%)** | **Uncertainty (CoV) (%)** | **Weight in Global Variance (%)** |
| --- | --- | --- | --- |
| Total released volume (M m3) | 72% | 63% | 90% |
| Yield Strength (Pa) | 5% | 9% | 2% |
| Viscosity (Pa.s) | 0% | 0% | 0% |
| Roughness | 19% | 18% | 7% |
| Top Breach Width (m) | 4% | 6% | 1% |

Table B‑36 Estimated sensitivity, uncertainty and weight in global variance of modelled flow front arrival time at 50% runout with respect to the listed inputs for the Tonglvshan TDB, China.

| **Input** | **Sensitivity Measurement (%)** | **Uncertainty (CoV) (%)** | **Weight in Global Variance (%)** |
| --- | --- | --- | --- |
| Total released volume (M m3) | 3% | 2% | 0% |
| Yield Strength (Pa) | 3% | 4% | 0% |
| Viscosity (Pa.s) | 3% | 1% | 0% |
| Roughness | 42% | 30% | 23% |
| Top Breach Width (m) | 48% | 55% | 77% |

Table B‑37 Estimated sensitivity, uncertainty and weight in global variance of modelled inundation area with respect to the listed inputs for the Cadia Event 2 TDB, Australia.

| **Input** | **Sensitivity Measurement (%)** | **Uncertainty (CoV) (%)** | **Weight in Global Variance (%)** |
| --- | --- | --- | --- |
| Total released volume (M m3) | 54% | 43% | 81% |
| Yield Strength (Pa) | 32% | 15% | 9% |
| Viscosity (Pa.s) | 3% | 2% | 0% |
| Roughness | 0% | 0% | 0% |
| Top Breach Width (m) | 11% | 15% | 9% |

Table B‑38 Estimated sensitivity, uncertainty and weight in global variance of modelled maximum flow velocity at 50% runout with respect to the listed inputs for the Cadia Event 2 TDB, Australia.

| **Input** | **Sensitivity Measurement (%)** | **Uncertainty (CoV) (%)** | **Weight in Global Variance (%)** |
| --- | --- | --- | --- |
| Total released volume (M m3) | 38% | 90% | 34% |
| Yield Strength (Pa) | 9% | 13% | 1% |
| Viscosity (Pa.s) | 19% | 32% | 4% |
| Roughness | 4% | 9% | 0% |
| Top Breach Width (m) | 30% | 120% | 61% |

Table B‑39 Estimated sensitivity, uncertainty and weight in global variance of modelled maximum flow depth at 50% runout with respect to the listed inputs for the Cadia Event 2 TDB, Australia.

| **Input** | **Sensitivity Measurement (%)** | **Uncertainty (CoV) (%)** | **Weight in Global Variance (%)** |
| --- | --- | --- | --- |
| Total released volume (M m3) | 40% | 22% | 20% |
| Yield Strength (Pa) | 0% | 0% | 0% |
| Viscosity (Pa.s) | 9% | 4% | 1% |
| Roughness | 5% | 3% | 0% |
| Top Breach Width (m) | 46% | 43% | 79% |

Table B‑40 Estimated sensitivity, uncertainty and weight in global variance of modelled flow front arrival time at 50% runout with respect to the listed inputs for the Cadia Event 2 TDB, Australia.

| **Input** | **Sensitivity Measurement (%)** | **Uncertainty (CoV) (%)** | **Weight in Global Variance (%)** |
| --- | --- | --- | --- |
| Total released volume (M m3) | 13% | 10% | 2% |
| Yield Strength (Pa) | 13% | 6% | 1% |
| Viscosity (Pa.s) | 25% | 14% | 4% |
| Roughness | 0% | 0% | 0% |
| Top Breach Width (m) | 50% | 67% | 93% |

Table B‑41 Estimated sensitivity, uncertainty and weight in global variance of modelled inundation area with respect to the listed inputs for the Feijão TDB, Brazil.

| **Input** | **Sensitivity Measurement (%)** | **Uncertainty (CoV) (%)** | **Weight in Global Variance (%)** |
| --- | --- | --- | --- |
| Total released volume (M m3) | 56% | 30% | 56% |
| Yield Strength (Pa) | 10% | 12% | 8% |
| Viscosity (Pa.s) | 0% | 0% | 0% |
| Roughness | 7% | 4% | 1% |
| Top Breach Width (m) | 26% | 24% | 35% |

Table B‑42 Estimated sensitivity, uncertainty and weight in global variance of modelled maximum flow velocity at 50% runout with respect to the listed inputs for the Feijão TDB, Brazil.

| **Input** | **Sensitivity Measurement (%)** | **Uncertainty (CoV) (%)** | **Weight in Global Variance (%)** |
| --- | --- | --- | --- |
| Total released volume (M m3) | 37% | 40% | 37% |
| Yield Strength (Pa) | 6% | 15% | 6% |
| Viscosity (Pa.s) | 1% | 0% | 1% |
| Roughness | 53% | 62% | 53% |
| Top Breach Width (m) | 3% | 6% | 3% |

Table B‑43 Estimated sensitivity, uncertainty and weight in global variance of modelled maximum flow depth at 50% runout with respect to the listed inputs for the Feijão TDB, Brazil.

| **Input** | **Sensitivity Measurement (%)** | **Uncertainty (CoV) (%)** | **Weight in Global Variance (%)** |
| --- | --- | --- | --- |
| Total released volume (M m3) | 85% | 39% | 97% |
| Yield Strength (Pa) | 4% | 4% | 1% |
| Viscosity (Pa.s) | 0% | 0% | 0% |
| Roughness | 9% | 4% | 1% |
| Top Breach Width (m) | 2% | 2% | 0% |

Table B‑44 Estimated sensitivity, uncertainty and weight in global variance of modelled flow front arrival time at 50% runout with respect to the listed inputs for the Feijão TDB, Brazil.

| **Input** | **Sensitivity Measurement (%)** | **Uncertainty (CoV) (%)** | **Weight in Global Variance (%)** |
| --- | --- | --- | --- |
| Total released volume (M m3) | 29% | 25% | 19% |
| Yield Strength (Pa) | 5% | 9% | 2% |
| Viscosity (Pa.s) | 1% | 0% | 0% |
| Roughness | 48% | 44% | 59% |
| Top Breach Width (m) | 17% | 26% | 20% |

- Tables for the Sensitivity Variation Along Runout Path

Table C‑1 Variation of sensitivity with distance from the breach for modelled maximum flow velocity for the Stava TDB, Italy

| % of Runout Distance | TRV | YS | V | R | BW |
| --- | --- | --- | --- | --- | --- |
| 10 | 4% | 0% | 0% | 61% | 35% |
| 25 | 18% | 1% | 1% | 58% | 22% |
| 50 | 27% | 0% | 0% | 60% | 12% |
| 75 | 26% | 0% | 0% | 67% | 7% |
| 90 | 34% | 0% | 1% | 58% | 6% |

Table C‑2 Variation of sensitivity with distance from the breach for modelled maximum flow depth for the Stava TDB, Italy

| % of Runout Distance | TRV | YS | V | R | BW |
| --- | --- | --- | --- | --- | --- |
| 10 | 1% | 0% | 2% | 5% | 92% |
| 25 | 17% | 0% | 0% | 55% | 28% |
| 50 | 30% | 0% | 1% | 53% | 16% |
| 75 | 65% | 0% | 0% | 23% | 12% |
| 90 | 85% | 0% | 0% | 12% | 4% |

Table C‑3 Variation of sensitivity with distance from the breach for modelled flow front arrival time for the Stava TDB, Italy

| % of Runout Distance | TRV | YS | V | R | BW |
| --- | --- | --- | --- | --- | --- |
| 10 | 0% | 0% | 0% | 0% | 100% |
| 25 | 7% | 0% | 0% | 50% | 43% |
| 50 | 16% | 0% | 0% | 53% | 31% |
| 75 | 23% | 0% | 0% | 57% | 21% |
| 90 | 23% | 0% | 0% | 59% | 17% |

Table C‑4 Variation of sensitivity with distance from the breach for modelled maximum flow velocity for the Aznalcóllar TDB, Spain

| % of Runout Distance | TRV | YS | V | R | BW |
| --- | --- | --- | --- | --- | --- |
| 10 | 7% | 0% | 0% | 41% | 30% |
| 25 | 21% | 0% | 0% | 59% | 12% |
| 50 | 8% | 0% | 0% | 88% | 4% |
| 75 | 26% | 0% | 0% | 74% | 0% |
| 90 | 21% | 0% | 0% | 71% | 0% |

Table C‑5 Variation of sensitivity with distance from the breach for modelled maximum flow depth for the Aznalcóllar TDB, Spain

| % of Runout Distance | TRV | YS | V | R | BW |
| --- | --- | --- | --- | --- | --- |
| 10 | 47% | 0% | 0% | 21% | 19% |
| 25 | 71% | 0% | 0% | 11% | 11% |
| 50 | 88% | 0% | 0% | 8% | 4% |
| 75 | 100% | 0% | 0% | 0% | 0% |
| 90 | 95% | 0% | 0% | 5% | 0% |

Table C‑6 Variation of sensitivity with distance from the breach for modelled flow front arrival time for the Aznalcóllar TDB, Spain

| % of Runout Distance | TRV | YS | V | R | BW |
| --- | --- | --- | --- | --- | --- |
| 10 | 8% | 0% | 0% | 52% | 20% |
| 25 | 7% | 0% | 0% | 42% | 30% |
| 50 | 15% | 0% | 0% | 50% | 21% |
| 75 | 13% | 0% | 1% | 62% | 14% |
| 90 | 13% | 0% | 1% | 66% | 12% |

Table C‑7 Variation of sensitivity with distance from the breach for modelled maximum flow velocity for the Feijão TDB, Brazil maximum flow depth, and frontal arrival time

| % of Runout Distance | TRV | YS | V | R | BW |
| --- | --- | --- | --- | --- | --- |
| 10 | 18% | 1% | 0% | 52% | 30% |
| 25 | 23% | 2% | 0% | 53% | 23% |
| 50 | 37% | 6% | 1% | 53% | 3% |
| 75 | 38% | 12% | 1% | 49% | 0% |
| 90 | 33% | 14% | 1% | 51% | 1% |

Table C‑8 Variation of sensitivity with distance from the breach for modelled maximum flow depth for the Feijão TDB, Brazil

| % of Runout Distance | TRV | YS | V | R | BW |
| --- | --- | --- | --- | --- | --- |
| 10 | 41% | 1% | 0% | 1% | 57% |
| 25 | 75% | 4% | 0% | 7% | 14% |
| 50 | 85% | 4% | 0% | 9% | 2% |
| 75 | 85% | 11% | 1% | 3% | 0% |
| 90 | 83% | 12% | 0% | 4% | 1% |

Table C‑9 Variation of sensitivity with distance from the breach for modelled flow front arrival time for the Feijão TDB, Brazil

| % of Runout Distance | TRV | YS | V | R | BW |
| --- | --- | --- | --- | --- | --- |
| 10 | 11% | 0% | 11% | 22% | 56% |
| 25 | 19% | 3% | 0% | 41% | 38% |
| 50 | 29% | 5% | 1% | 48% | 17% |
| 75 | 37% | 9% | 2% | 42% | 9% |
| 90 | 39% | 11% | 1% | 42% | 7% |
